# Response surface optimization of sedimentation efficiency for sustainable green microalgae harvesting using automated non-invasive methods

**DOI:** 10.1186/s12934-025-02765-2

**Published:** 2025-07-11

**Authors:** Amr M. Ayyad, Eladl G. Eltanahy, Mervat H. Hussien, Dina A. Refaay

**Affiliations:** Botany Department, Faculty of Science, Mansoura University, Mansoura, 35516 Egypt

**Keywords:** *Chlorella sorokiniana*, *Monoraphidium convolutum*, Response surface methodology optimization, Central composite design, Eco-friendly passive harvesting, Microalgae sedimentation, Automated microimaging, Non-invasive measurements, Advanced photographic measurement

## Abstract

**Background:**

Microalgae such as *Chlorella sorokiniana* and *Monoraphidium convolutum* are promising sources for biofuels, pharmaceuticals, nutraceuticals, and wastewater treatment. However, biomass harvesting remains a cost-intensive bottleneck. Conventional methods like centrifugation and flocculation pose challenges due to energy demands and contamination risks. Sedimentation offers a passive, eco-friendly alternative but is highly sensitive to environmental and physiological variables. This study integrates response surface methodology with a novel, non-invasive photographic imaging technique to optimize sedimentation efficiency.

**Results:**

Both species exhibited optimal growth in Bold Basal Medium, achieving cell densities of 29.59 and 9.5 million cells per mL, respectively. Automated cell counting strongly correlated with manual methods (R^2^ = 98.99%). Biochemical analysis revealed a higher protein content in *C. sorokiniana* (61.6%) and greater lipid content in *M. convolutum* (39.31%). Sedimentation efficiency was highest at acidic pH and low salinity, reaching 96.14% for *C. sorokiniana* and 88.7% for *M. convolutum*. Sealed vessels and smaller culture volumes further enhanced sedimentation efficiency. RSM predictive models achieved high accuracy (adjusted R^2^ > 99%). A novel, real-time photographic method for sedimentation assessment was introduced, offering a non-invasive, sampling-free alternative to conventional techniques. This method strongly correlated with OD-based measurements (R^2^ = 94.89%) and presents a scalable solution for continuous biomass monitoring. Compared to conventional centrifugation, the optimized sedimentation approach is estimated to reduce harvesting costs by 77–79%.

**Conclusions:**

This study advances sedimentation-based harvesting of *C. sorokiniana* and *M. convolutum* by integrating RSM with a novel, automated, non-invasive imaging technique for sedimentation monitoring. This approach, rarely applied in microalgae harvesting, enables real-time assessment without disturbing the culture, enhancing process control and scalability. Sedimentation efficiency was influenced by cell morphology, biochemical composition, and environmental factors such as pH, salinity, gas exchange, and culture volume. The optimized conditions not only improved harvesting precision and reproducibility but also reduced harvesting costs, highlighting the method’s potential for economic and environmentally sustainable deployment in large-scale microalgae-based production systems for biofuels, bioplastics, and high-value compounds.

**Graphical Abstract:**

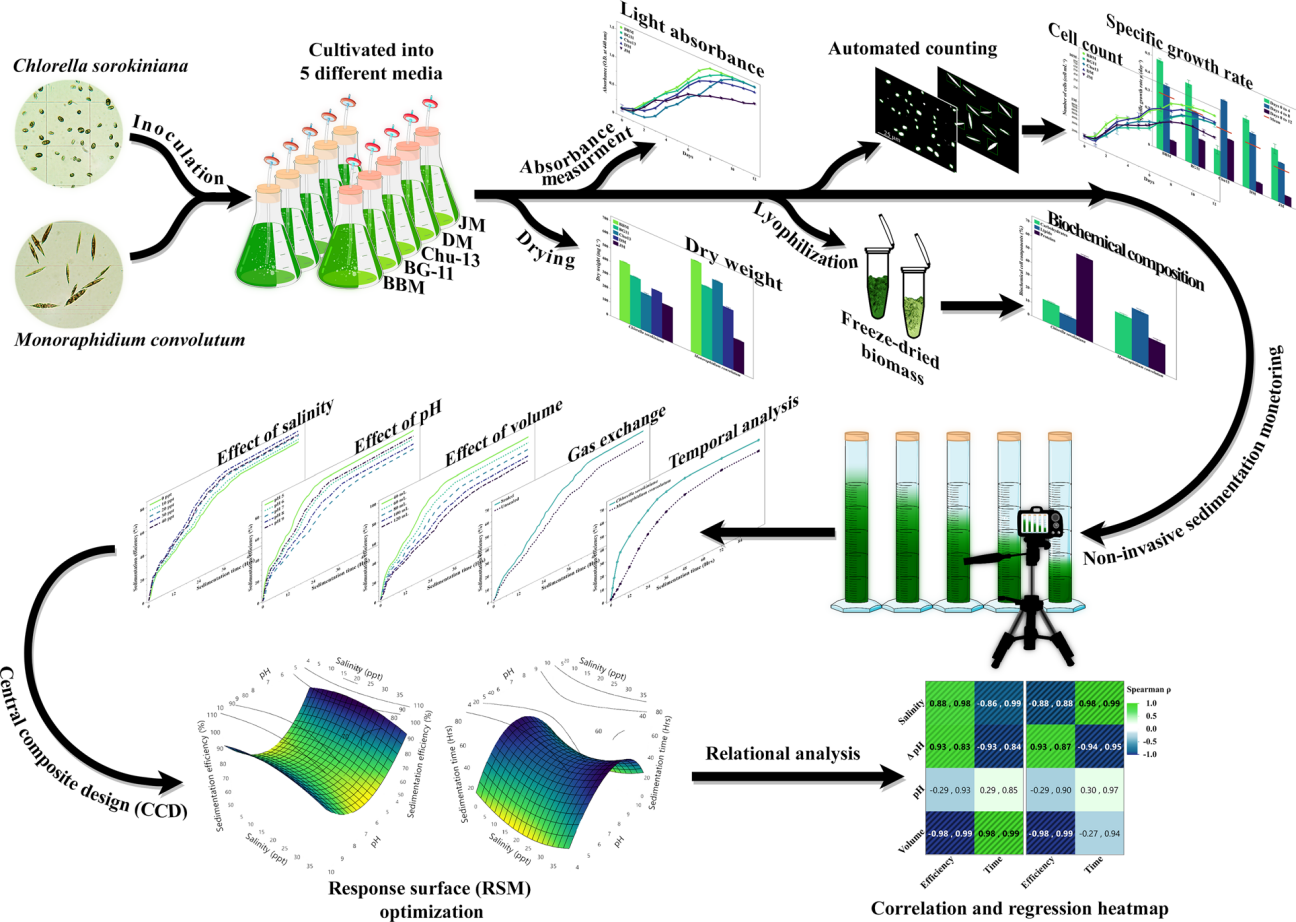

## Background

Microalgae have emerged as a versatile and sustainable resource with wide-ranging applications in biofuels, pharmaceuticals, nutraceuticals, and wastewater treatment. These photosynthetic microorganisms are known for their rapid growth rates and high lipid, protein, and carbohydrate contents, making them ideal candidates for numerous industrial processes [1, 2].

Furthermore, microalgae can be readily cultivated under controlled laboratory conditions, allowing precise regulation of parameters such as light intensity, temperature, aeration, and nutrient composition. Standardized culture media, such as Bold’s Basal Medium (BBM), provide consistent nutrient profiles that support reproducible biomass production across diverse species. These controlled conditions are essential for studying species-specific responses to environmental variables and optimizing downstream processes such as harvesting efficiency [3].

Among the numerous species of microalgae, green microalgae such as *Chlorella sorokiniana* and *Monoraphidium convolutum* are particularly noteworthy. *C. sorokiniana* is highly valued in the pharmaceutical industry due to its rich nutrient profile, including essential vitamins, minerals, and antioxidants [4–7]. It has been explored for its potential in boosting immune function, detoxification, and even cancer prevention. On the other hand, *M. convolutum* has shown significant promise in biofuel production. Its high lipid content and efficient growth make it an excellent candidate for producing biofuels [8–10]. This species has also been studied for its role in bioremediation, where it helps in removing pollutants from wastewater, thus providing an environmentally friendly solution to waste management [11, 12].

Despite their promising potential, one of the main hurdles in the industrial-scale cultivation of microalgae is the efficient and cost-effective harvesting of biomass. Conventional harvesting methods, such as flocculation and centrifugation, have significant drawbacks especially when scaled up for industrial use [13].

Centrifugation, although effective, is particularly energy-intensive and expensive. The operational costs associated with continuous centrifugation are prohibitively high, largely due to the costs of purchase and maintenance of the equipment needed for continuous centrifugation as well as the substantial electricity consumption required to maintain high-speed rotations [14]. This not only increases the overall production costs but also significantly raises the carbon footprint of the process, undermining the sustainability of microalgae-based products.

Flocculation, on the other hand, often requires the addition of chemical agents like aluminum and iron salts to aggregate the microalgal cells, making it easier to separate the cells from the growth medium. These chemicals can introduce toxic residues, leading to contamination of the biomass, complicating downstream processing, and making it unsuitable for applications where purity is essential, such as in pharmaceuticals [15]. Furthermore, these chemical additives can reduce the quality of biofuels produced, leading to inefficiencies and additional processing requirements [16]. Additionally, the environmental impact of chemical waste is a concern, further detracting from the sustainability of this method.

Sedimentation offers a promising natural and eco-friendly alternative to these traditional harvesting methods. This process relies on gravity to settle the microalgal cells from the culture medium, eliminating the need for chemical additives and reducing energy costs, and can be implemented using simple and inexpensive equipment [17]. It is particularly attractive for its simplicity and potential for scalability, where cost and sustainability are critical considerations. Additionally, sedimentation produces nontoxic products, ensuring the purity of the harvested biomass, aligning well with the growing demand for environmentally responsible and sustainable industrial practices. However, despite its advantages in terms of simplicity and energy efficiency, the efficiency of sedimentation is generally lower than other harvesting methods and can be significantly influenced by various factors. These include environmental and physiological parameters such as pH, ionic strength, and gas exchange, as well as the physical properties of the culture, column height, and the specific characteristics of the microalgal strain, cell morphology and growth medium composition. These factors collectively limit the standalone applicability of sedimentation without appropriate optimization [18].

Manual counting of microalgal cells is a labor-intensive and time-consuming process and prone to inconsistency, particularly when handling large sample volumes [19, 20]. Recent advances in automated counting methods, such as those utilizing machine learning models for cell detection [21], have revolutionized this aspect of microalgae research. These techniques drastically reduce the time and manpower required, enabling high-throughput data collection with enhanced precision and consistency. By streamlining the process, automated methods free up resources for other critical research activities while minimizing human error.

Assessing sedimentation trends, including efficiency and time, is another critical component of harvesting optimization. Traditional methods relying on spectrophotometric absorbance can be labor-intensive and require invasive sampling [22], which risks disrupting the sedimentation process and altering the stability of the vessel. Newer techniques, such as acoustic monitoring [23], provide a more comprehensive and less invasive method for monitoring suspended sediment in water bodies. In contrast to traditional methods, non-invasive photographic methods allow for real-time monitoring of sedimentation without disturbing the system. This approach not only preserves the integrity of the sedimentation process but also provides continuous, high-resolution data, making it a superior alternative for assessing sedimentation dynamics.

The application of Design of Experiments (DoE), particularly Response Surface Methodology (RSM), has emerged as a robust strategy for optimizing experimental variables [24]. By systematically evaluating the interactions between factors, RSM reduces the need for excessive resource allocation while delivering precise and actionable data. This methodology enables researchers to identify optimal conditions with fewer experimental runs, ensuring the efficient use of time, materials, and labor [25].

These methodologies align closely with the goals of sustainability in microalgae research. By reducing the time, resources, and energy required for critical processes such as cell counting, sedimentation monitoring, and experimental optimization, they offer practical solutions to some of the major bottlenecks in industrial-scale production. Furthermore, the integration of eco-friendly and non-invasive techniques underscores the commitment to minimizing environmental impact while ensuring the efficiency and scalability of microalgal harvesting methods.

Broader implications of the study suggest its applicability in large-scale production. The innovations discussed not only enhance laboratory-scale research but also provide a framework for scaling up microalgae cultivation and harvesting processes. The use of advanced imaging and automated methodologies minimizes human intervention, while optimized harvesting strategies ensure that industrial operations can be both cost-effective and sustainable. These advancements pave the way for the commercialization of microalgae-based products, addressing critical challenges in energy production, environmental conservation, and bioeconomy.

The objectives of this study were to evaluate the growth performance of *C. sorokiniana* and *M. convolutum* in different growth media and to assess the sedimentation efficiency under various conditions. The specific goals were to identify the optimal growth medium and harvest time, determine the effects of time, gas exchange, and column volume on sedimentation efficiency, followed by optimization via Response Surface Methodology, and utilize advanced imaging techniques for non-invasive quantification of sedimentation.

## Methods

### Microalgae species

Two species of green freshwater microalgae, *Chlorella sorokiniana* (MZ348902.1) and *Monoraphidium convolutum* (OQ420741.1), were obtained from the Mansoura University Algae Biotechnology Lab algal collection. These strains were originally isolated from freshwater environments and were both morphologically and molecularly identified [26, 27]. They were selected for this study based on their contrasting morphological and biochemical traits, *C. sorokiniana* being protein-rich and spherical, and *M. convolutum* being lipid-rich and ellipsoidal, which provide a meaningful basis for evaluating species-specific responses to sedimentation conditions. Their well-documented industrial relevance in feed, pharmaceuticals, and biofuel applications further supports their suitability as model organisms for optimizing eco-friendly harvesting techniques [7, 8, 28].

### Evaluation of growth metrics and media selection

Five different growth media were evaluated to determine the optimal medium for microalgae growth: Bold’s Basal Medium (BBM) [29], BG-11 Medium (BG11) [30], Jaworski Medium (JM) [31], Chu 13 Medium (Chu13) [32], and Diatoms Medium (DM) [33]. Each medium was prepared according to standard protocols and sterilized by autoclaving at 121 °C for 20 min to avoid contamination.

To recognize the optimal growth medium and harvest time, each species was cultivated in each of the five media. Growth was monitored daily using light absorbance and cell counts and dry weight measurements were done in a separate experiment. The medium and time point yielding the highest significant cell density, cell concentration and dry weight were considered optimal.

Culturing was started from stock inocula maintained under optimal conditions. The inoculum age was standardized to ensure consistency in the initial growth phase. Each experimental setup consisted of 500 mL Erlenmeyer flasks containing 400 mL of the respective growth medium. Cultures were maintained under controlled laboratory conditions. Light intensity was set at 100 μmol photons m^−2^ s^−1^ with a photoperiod of 16:8 h light–dark cycle. The temperature was kept constant at 25 ± 1 °C. To ensure homogeneity and prevent sedimentation during growth, cultures were continuously aerated with filtered air using air pumps equipped with 0.22 μm pore size filters. [34].

#### Spectrophotometric absorbance measurement

The growth of both microalgae species was monitored daily by measuring the optical density (OD) of the culture at 440 nm using a spectrophotometer [35, 36]. Samples were diluted when necessary to keep readings within the linear range of the instrument [37]. The OD_440nm_ values were plotted against time to monitor growth and determine the most suitable time for harvesting for each microalgal species [38].

#### Automated cell counting through microimaging

**Fig. 1:**
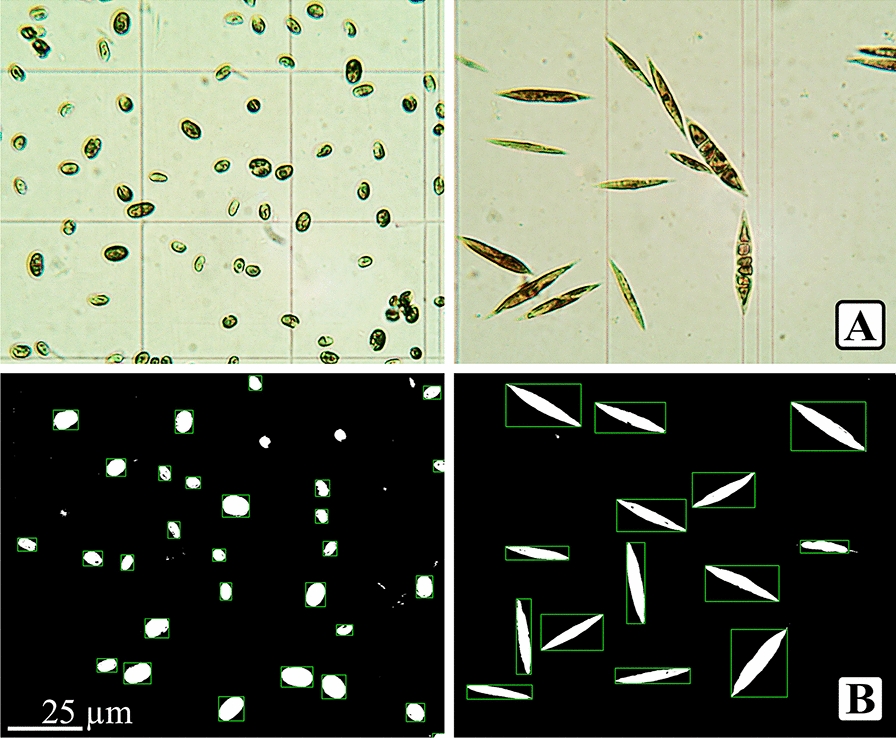
Microscopic view of microalgal cells on a hemocytometer slide at 400× magnification (**A**). Processed version for enhanced isolation and final automated recognition and counting of cells (**B**)

Daily samples were taken and fixed with Lugol's iodine solution, ensuring the fixation of the samples and their distinct appearance under the microscope. The samples were loaded into a hemocytometer, and images were captured at 100× and 400× magnification to determine cell number, size, and morphology using a microscopy setup [39]. The captured images were then processed using Fiji 2.16.0 [40] and Python 3.12.3 scripting language [41] with the Open Computer Vision library (OpenCV) [42], utilizing watershed image segmentation algorithms for automated cell detection (as shown in Fig. 1). Notably, this approach does not rely on any custom machine learning models, unlike previous studies [21], making it an open-access and easy-to-apply method that significantly reduces processing time.

To validate the accuracy of the automated counting technique, a stratified random sampling approach was used to select 10% of the images. These images were manually counted, and the results were cross-referenced with the automated counts, assessing the reliability and consistency between the two methods.

The specific growth rate (µ) of each microalga was calculated using the equation of Guillard [43] (Eq. 1):

1 $$Specific\; growth\; rate \left(\mu \right) = \text{ } \frac{\text{ln}\left({n}_{2}\right)-\text{ln}\left({n}_{1}\right)}{{t}_{2}-{t}_{1}}$$

where (${n}_{1}$) represents the initial cell concentration at time (${t}_{1}$), and (${n}_{2}$) represents the cell concentration at a later time (${t}_{2}$). The difference between (${t}_{2}$) and (${t}_{1}$) represents the duration of the exponential growth phase. The calculation of specific growth rates was performed across intervals (e.g., 0–4, 4–8, 8–12 days), and the average specific growth rate was then determined using the following equation (Eq. 2):

2 $$Average\; specific\; growth\; rate \left({\mu }_{avg}\right)= \frac{{\sum }_{i=1}^{n} {\mu }_{i }}{n}$$

where (${\mu }_{i}$) is the specific growth rate for each interval (*i*) and ($n$) is the total number of intervals. This interval-based approach ensures greater reliability in calculating specific growth rates and enables the investigation of growth dynamics during each time interval [44, 45].

#### Quantitative analysis of dry biomass

Dry biomass was measured using an independent experimental setup to ensure sufficient material for accurate gravimetric analysis. While growth metrics such as optical density and cell count were conducted in 500 mL Erlenmeyer flasks with 400 mL working volume, dry biomass determinations were performed in larger 1000 mL flasks containing 800 mL of the same growth medium. These setups were conducted in and maintained under identical culture conditions, with samples harvested at the previously determined optimal growth phase for each species to ensure consistency across measurements. At the optimum harvest time for each species, samples were filtered using pre-weighed Whatman GF/C filters. The filters with biomass were then dried at 60 °C until a constant weight was achieved in a humidity-controlled oven and reweighed to determine the dry weight (mg L^−1^) of the microalgae biomass [38].

#### Biochemical cell components analysis

Based on the growth performance outcomes of the two microalgae tested, BBM was selected as the optimal candidate nutrient medium for further analyses, beginning with the evaluation of biochemical cell components. Total carbohydrate content was measured using the phenol–sulfuric acid method described by Dubois et al. [46]. Briefly, a known weight of lyophilized algal biomass was hydrolyzed by 2.5 N HCl in a boiling water bath. After neutralization, total volume was adjusted with dist. Water and then treated with 5% phenol solution, followed by the addition of concentrated sulfuric acid. The reaction mixture was incubated at room temperature for 30 min, and absorbance was measured at 490 nm using a UV–Vis spectrophotometer. A glucose standard curve was used for calibration.

Lipid content was assessed gravimetrically following the Bligh and Dyer method [47]. Algal lyophilized biomass was mixed with a 2:1 (v/v) chloroform–methanol solution, vortexed, and incubated at room temperature till sufficient extraction. To separate the extract into layers, 0.9 N NaCl was added. The organic phase, containing lipids, was separated, evaporated, and the lipid content was determined by weighing the remaining residue.

Finally, protein content was estimated following the Lowry et al. [48], with crystalline bovine serum albumin (BSA) as the standard. Lyophilized biomass was homogenized with phosphate buffer (pH 7), then reacted with alkaline copper sulfate solution, followed by the addition of Folin–Ciocalteu reagent. After incubation for 30 min at room temperature, absorbance was measured at 750 nm.

### Investigation of sedimentation efficiency

A series of sedimentation experiments was conducted to evaluate the sedimentation efficiency and time under the effect of different conditions. Each experiment commenced with the acquisition of microalgal cultures at their respective optimal harvest time. The sizes and volumes utilized were selected to ensure practical feasibility and alignment with available resources, enhancing the reliability and reproducibility of the method.

#### Temporal analysis of sedimentation

This experiment investigated the effect of sedimentation time on efficiency. Equal volumes (100 mL) of an equal concentration of cultured microalgae were transferred into separate containers. The containers were labeled and harvested at specific intervals (0, 1, 2, 4, 6, 12, 18, 24, 30, 36, 48, 72, and 96 h). At each timepoint, samples were taken from the middle height of the container using a sterile pipette to ensure consistency.

Sedimentation efficiency measuring using optical density (SE_OD_) was calculated by comparing the biomass concentration in the supernatant with the initial concentration, using light absorbance at 440 nm wavelength. The calculations were done using the following equation (Eq. 3):

3 $${Sedimentation\; efficiency}_{OD} \left(\%\right)=\frac{{A}_{0}-{A}_{t}}{{A}_{0}}\times 100$$

where (${A}_{0}$) indicates the Initial absorbance prior to sedimentation and absorbance after sedimentation time was denoted by (${A}_{t}$).

#### Influence of gas exchange on sedimentation efficiency

According to the previous experiment results, the maximum time needed to achieve the highest significant values of sedimentation efficiency was 72 h. Consequently, the subsequent experiments focused on studying the different parameters of sedimentation within this timeframe. The primary goal of these experiments was to compare results within the allotted time rather than to extend the documentation duration.

The impact of gas exchange on sedimentation efficiency was examined using multiple graduated cylinders. Half of the cylinders were covered to restrict gas exchange, and the other half left open to the air. Each column was filled with an equal concentration of microalgae culture. By comparing sedimentation in sealed versus open environments, this experiment evaluated the impact of gas exchange on sedimentation efficiency. The sedimentation efficiency measured using green color intensity (SE_GCI_) of the cultures was then quantified through a non-invasive photography technique and color analysis using Fiji software and Python scripts.

#### Effect of culture column volume on sedimentation

The influence of column volume on sedimentation efficiency was studied using graduated cylinders of the same size and labeled according to each varying volume (40, 60, 80, 100, 120 mL). Each cylinder was filled with an equal concentration of microalgae culture and monitored at the same time continuously using non-invasive photography techniques similar to the prior experiment to ensure that the sedimentation efficiency was evaluated over time.

#### Impact of pH variation on sedimentation dynamics

The effect of pH on sedimentation efficiency was investigated by adjusting the pH level of the cultures to five levels: 5, 6, 7, 8, and 9. To ensure consistency, each pH level was tested in triplicate graduated cylinders after adjusting the pH using 0.1 N NaOH or 0.1 N HCl. The cylinders were labeled and filled with an equal concentration of microalgae culture in accordance with the tested pH levels and monitored at the same time continuously using the same non-invasive technique as the prior experiment to ensure that the sedimentation efficiency was properly evaluated.

#### Role of salinity on sedimentation efficiency

Salinity effect on sedimentation efficiency was investigated by directly dissolving NaCl into the microalgae cultures immediately before the settling process. Salinity levels were adjusted to 0, 10, 20, 30, and 40 ppt. The appropriate added amounts of NaCl were weighed and mixed thoroughly into the cultures immediately prior to the settling phase.

### Sedimentation optimization through response surface methodology (RSM) using central composite design (CCD)

To evaluate the impact of pH and salinity on sedimentation efficiency and time, a Central Composite Design (CCD) was employed within the framework of Response Surface Methodology (RSM). The experimental design incorporated two independent variables: pH (Factor A) and salinity in ppt (Factor B), with two dependent outcomes: sedimentation efficiency and time. A 14-run matrix was constructed and divided into two blocks to ensure practical applicability.

The experimental setup utilized a 5-level full factorial design for the independent variables, as detailed in Table 1:

**Table 1:** Coded and actual level values of the experimental variables used for the response surface central composite design (CCD) matrix for both tested microalgae

Factor	Name	Level				
		−2	−1	0	+1	+2
A	pH	4	5.5	7	8.5	10
B	Salinity (ppt)	5	12.5	20	27.5	35

The point distribution for the CCD design was defined as 4 cube points, 3 center points in cube, 4 axial points and 3 center points in axial. The experimental data were analyzed using the response surface regression method and fitted to the following second-order quadratic polynomial equation (Eq. 4) [49, 50]:

4 $$\text{Y}= {\beta }_{0}+ \sum_{i=1}^{k}{\beta }_{i}{X}_{i} + \sum_{i=1}^{k}{\beta }_{ii}{X}_{I}^{2}+ \sum_{i<j}{\beta }_{ij}{ X}_{i} {X}_{j}+\varepsilon$$

where the response variable (Y) represents the dependent variable being studied, while ${\beta }_{0}$ is the intercept of the model. The coefficients of the linear terms are denoted as ${\beta }_{i}$, whereas ${\beta }_{ii}$ represents the coefficients of quadratic terms. Interaction effects between independent variables are captured by ${\beta }_{ij}$, with ${X}_{i}$ and ${X}_{i}$ representing the independent variables in the model. Lastly, $\varepsilon$ accounts for the random error.

Optimized points were derived from the model, identifying the ideal combination of pH and salinity for maximizing sedimentation efficiency while minimizing sedimentation time. These optimized points were subsequently validated by conducting experiments and comparing the predicted values to the observed results.

### Non-invasive photography-based sedimentation efficiency analysis

**Fig. 2:**
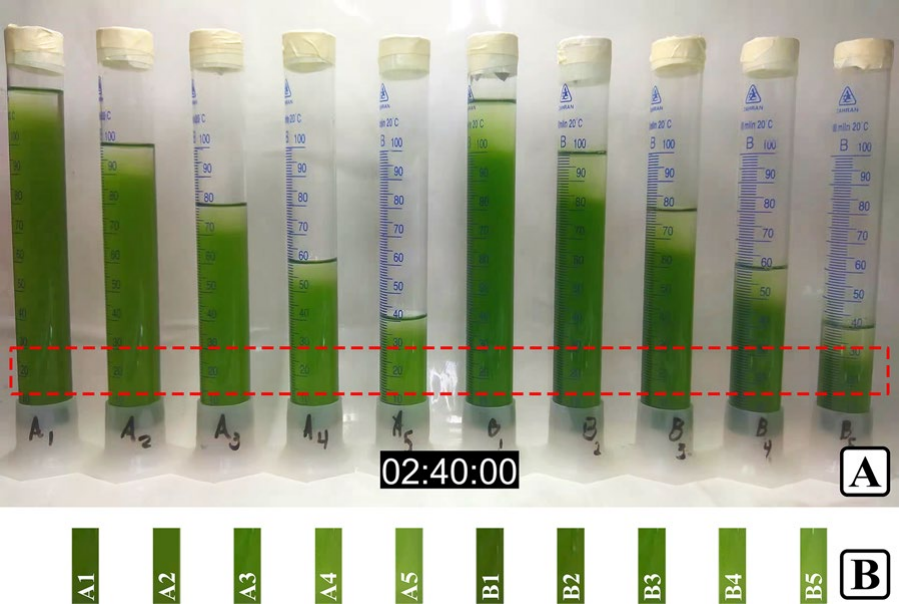
Time-lapse sequence capturing the sedimentation process highlighting the analysis area (**A**) and detailed view of the highlighted area segmentation (**B**)

To determine sedimentation efficiency in the second and later experiments, time-lapse captures were taken using a high-resolution camera with a relatively short time interval between each frame spanning the entire duration of the experiments (shown in Fig. 2) [51]. The images captured over the sedimentation period were segmented for each column at a constant measurement area. The intensity of the green color was analyzed using Fiji and Python scripts to determine the sedimentation efficiency (SE_GCI_), which was calculated according to the equation (Eq. 5):

5 $${Sedimentation\; efficiency}_{GCI} \left(\%\right)=\frac{{I}_{0}-{I}_{t}}{{I}_{0}}\times 100$$

where (${I}_{0}$) is the initial color intensity before sedimentation, and (${I}_{t}$) is the color intensity after a sedimentation time. This method allowed for precise quantification of sedimentation efficiency by measuring the decrease in green color intensity, indicative of microalgae settling over time all without disturbing the cultures.

At the beginning and end of each experiment, the optical density of the culture was measured to cross-reference the sedimentation efficiency calculated through both the manual (SE_OD_) and the non-invasive photographic (SE_GCI_) methods to validate the reliability of the technique.

### Statistical analysis

All experimental data collected were analyzed and visualized using JMP 18.1 [52] and Minitab 22.1 [53] statistical software. One-way analysis of variance (ANOVA) was conducted to evaluate the significance of differences in growth metrics and sedimentation efficiency. Post-hoc tests (Tukey’s HSD) and T-tests were employed to identify specific differences between group pairs. linear correlation coefficient (Pearson’s *r*) was calculated in cases of validation, and non-linear correlation coefficient (Spearmans’s *ρ*) in cases of factor-response relations. In addition to these methods, a Response Surface Methodology (RSM) approach was utilized to optimize both sedimentation efficiency and sedimentation time. A Central Composite Design (CCD) model comprising 14 experimental runs was constructed to explore the interactions between variables and identify optimal conditions. The RSM model analysis included the estimation of effects, ANOVA to assess model significance, and the use of a prediction profiler to visualize and predict optimal settings. Each experiment, excluding the methodology validation and CCD experimental trials, was conducted in triplicate to ensure statistical validity while maintaining resource feasibility, following standard practices in algal cultivation research. All experiments were analyzed using a 95% confidence interval. However, a 90% confidence interval was applied specifically to the RSM model. This choice reflects common practice in biological optimization studies to minimize the risk of overfitting, particularly given the limited number of experimental runs and the hypersensitivity of RSM models.

## Results

### Growth performance in various media

**Fig. 3:**
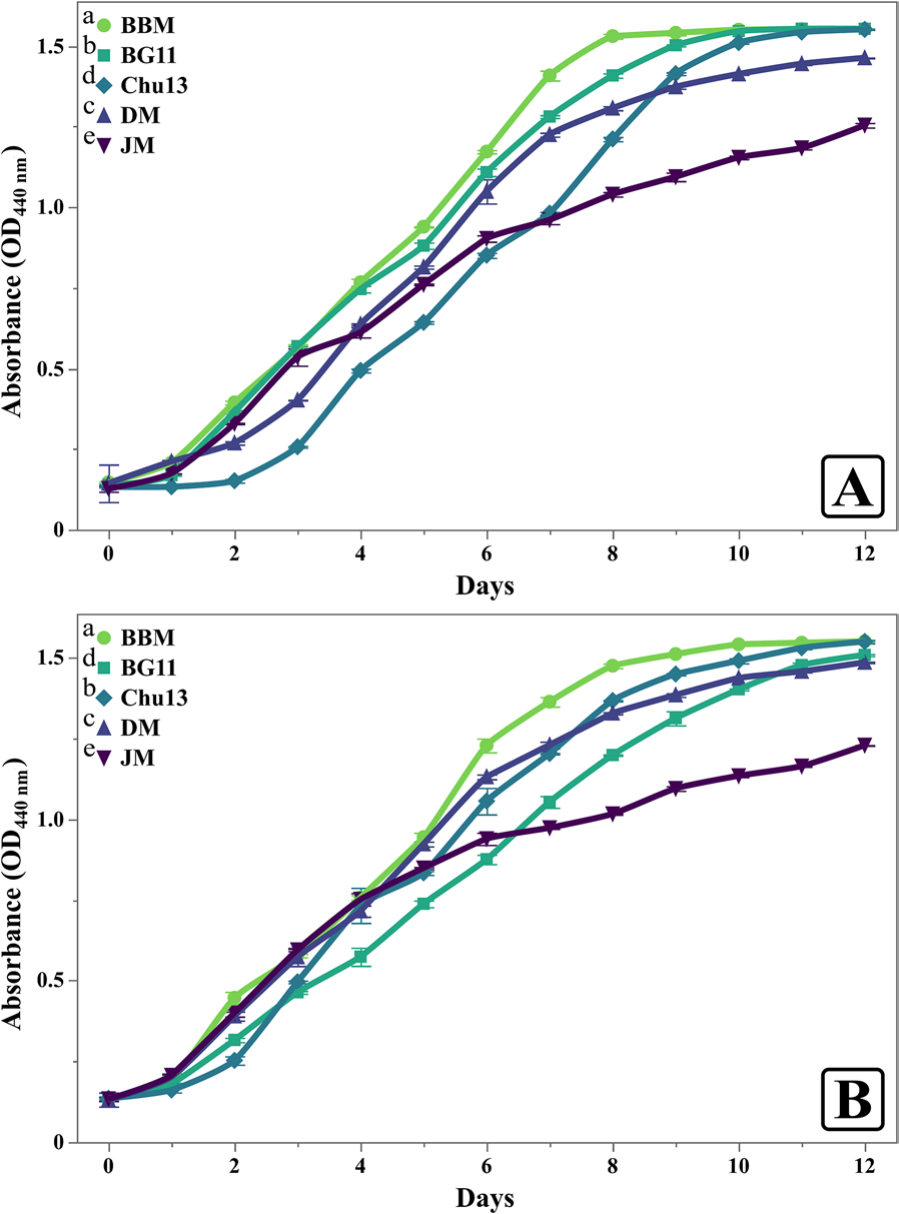
Light absorbance responses of *Chlorella sorokiniana* (**A**) and *Monoraphidium convolutum* (**B**) grown on different growth media, showcasing the higher values of BBM. Data is presented as means ± SE (n = 3). Letters report on legend indicate significant differences at harvest time

**Fig. 4:**
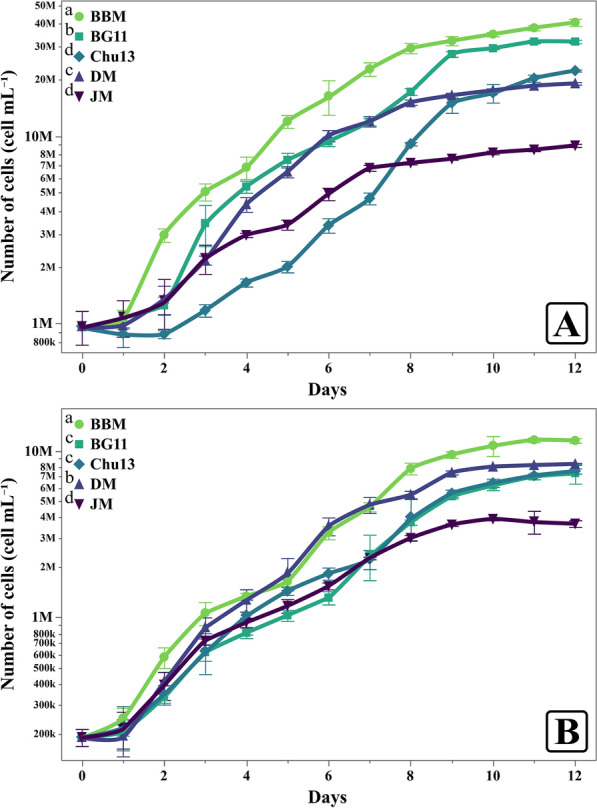
Daily cells count of *Chlorella sorokiniana* (**A**) and *Monoraphidium convolutum* (**B**) grown on different growth media with BBM resulting in higher cell counts. Data is presented as means ± SE (n = 3). Letters report on legend indicate significant differences at harvest time

**Fig. 5:**
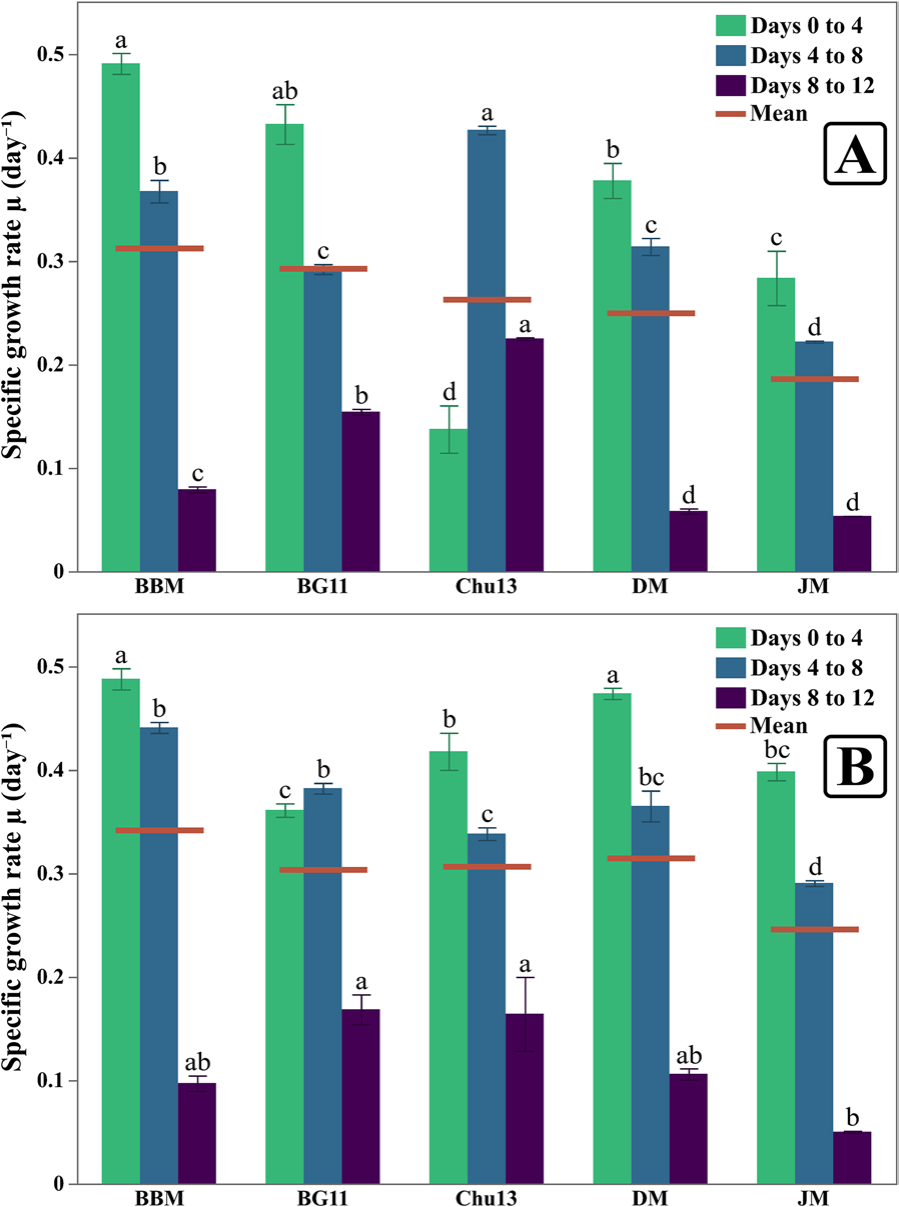
Specific growth rates of *Chlorella sorokiniana* (**A**) and *Monoraphidium convolutum* (**B**) in different growth media across different growth intervals and the mean specific growth rate for each medium. Data is presented as means ± SE (n = 3). Letters on error bars indicate significant differences in each interval

**Fig. 6:**
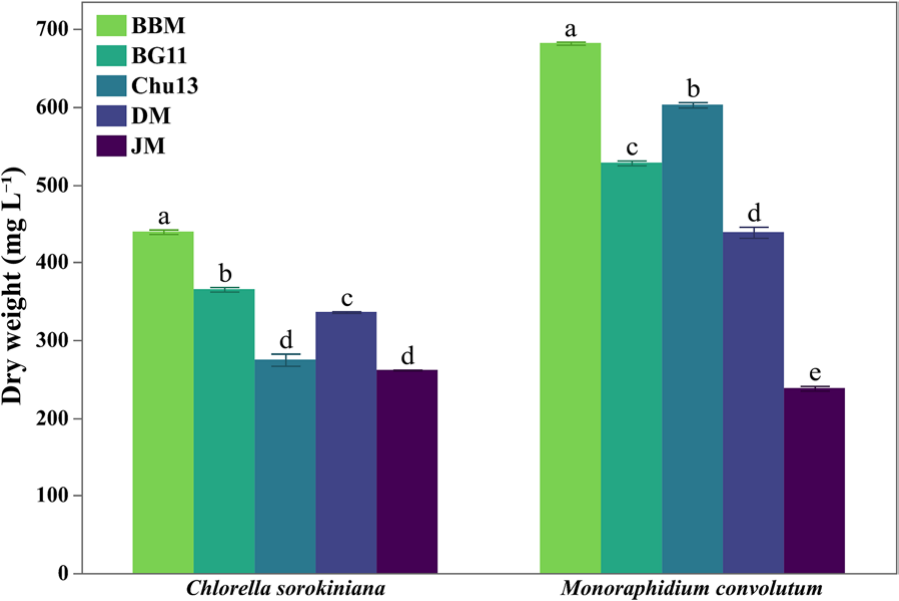
Dry weight investigation of both tested microalgal species at their respective harvest time of incubation in different growth media with BBM resulting in the higher weight for both species. Data is presented as means ± SE (n = 3). Letters on error bars indicate significant differences

The growth performance of *C. sorokiniana* and *M. convolutum* in response to the different growth media showed clear trends across culture density (OD_440nm_) (Fig. 3), number of cells (Fig. 4), specific growth rates (Fig. 5), and dry weight accumulation (Fig. 6).

For *C. sorokiniana*, BBM consistently demonstrated the highest growth across all parameters. Culture density in BBM reached 1.53 (Fig. 3A), also supported the highest cell density, reaching 29.59 million cells mL^−1^ at harvest time (Fig. 4A), significantly higher than the results obtained in other media. BBM also promoted the average specific growth rate of 0.31 day^−1^ (Fig. 5A). Finally, the dry weight of *C. sorokiniana* in BBM was 439.33 mg L^−1^ (Fig. 6), outperforming the results in other media when biomass production was observed. BG11 provided moderate support for growth. The light absorbance in BG11 was 1.408, lower than BBM but still relatively high compared to other media. The cell count in BG11 was 17.32 million cells mL^−1^, and the average specific growth rate was 0.29 day^−1^. The dry weight of *C. sorokiniana* in BG11 was 365.33 mg L^−1^, ranking second in biomass accumulation after BBM. Chu13 was less effective for *C. sorokiniana* growth, with OD_440nm_ of 1.211 and a cell count of only 9.13 million cells mL^−1^. The specific average growth rate for Chu13 was 0.26 day^−1^, and the dry weight was the third lowest at 274.67 mg L^−1^, indicating that this medium was not optimal for supporting high growth levels. Comparably, DM demonstrated moderate performance for *C. sorokiniana*. The OD_440nm_ was 1.307, and the cell count was 15.19 million cells mL^−1^. The average specific growth rate was 0.25 day^−1^, and the dry weight was 336.00 mg L^−1^, making DM a reasonable but not ideal medium for this microalga. JM, however, was the least effective medium. The OD_440nm_ was the lowest at 1.040, and the cell count reached only 7.22 million cells mL^−1^. The average specific growth rate was also the lowest at 0.19 day^−1^, with a corresponding dry weight of 261.33 mg L^−1^, indicating poor support for *C. sorokiniana* growth in this medium.

For *M. convolutum*, a similar trend was observed, with BBM promoting the highest growth across all measured parameters. The OD_440nm_ reached 1.511 (Fig. 3B) and the cell count was 9.5 million cells mL^−1^ (Fig. 4B), both the highest among the tested media. The average specific growth rate in BBM was 0.34 day^−1^ (Fig. 5B), and the dry weight was 682 mg L^−1^ (Fig. 6). Chu13 was the second most effective medium for *M. convolutum*, with an optical density of 1.449, a cell count of 5.57 million cells mL^−1^, and an average specific growth rate of 0.31 day^−1^. The dry weight in Chu13 was 602.67 mg L^−1^, making it a viable alternative to BBM for supporting *M. convolutum* growth. BG11 and DM displayed moderate results for *M. convolutum* growth. The optical density in BG11 was 1.313, with a cell count of 5.34 million cells mL^−1^. The average specific growth rate in BG11 was 0.3 day^−1^, and the dry weight was 528 mg L^−1^. DM showed a similar pattern, with an optical density of 1.385, a cell count of 7.41 million cells mL^−1^, and a specific growth rate of 0.31 day^−1^. The dry weight in DM was 438.67 mg L^−1^, indicating moderate but not optimal growth. JM was again the least suitable medium for *M. convolutum*, with the lowest optical density of 1.095, a cell count of 3.61 million cells mL^−1^, and the lowest average specific growth rate of 0.25 day^−1^. The dry weight in JM was also the lowest at 238 mg L^−1^, indicating poor performance for both test microalgae.

### Automated cell count validation

**Fig. 7:**
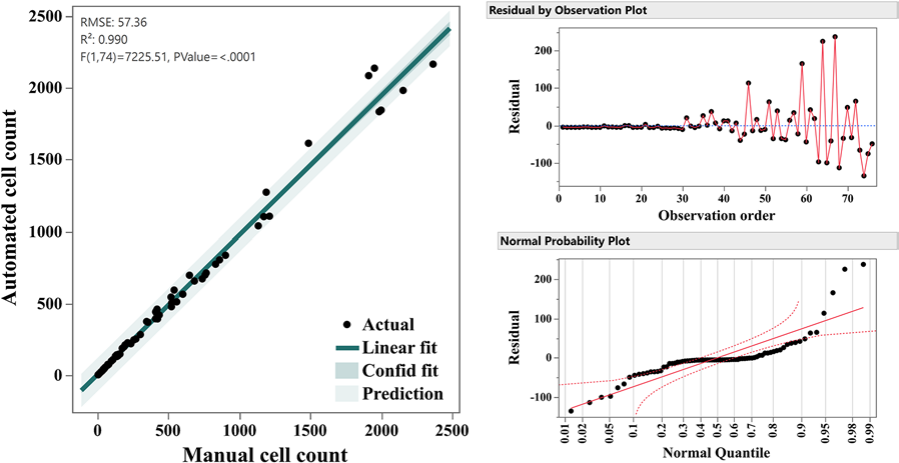
Comparative validation between automated and manual count methods using linear regression analysis (*left*), Residual analysis (*top-right*) signifies the general consistency of fit and normal probability plot (*bottom-right*) shows residuals distribution showcasing the significantly high accuracy of the method

The regression model analysis presented in Fig. 7 assesses the correlation between manual and automated counting methods in the stratified random 10%, sample amounting to 76 pictures. The resulting model demonstrated a high degree of accuracy, with an R^2^ value of 98.99%, the adjusted R^2^ value of 98.97% and the predicted R^2^ of 98.79% further affirmed the robustness of the model. The Pearson coefficient (*r*) between both methods (0.96) was statistically significant (*p*-value < 0.001), indicating a very strong positive linear relationship between the manual and automated counting methods.

### Biochemical composition of microalgae

**Fig. 8:**
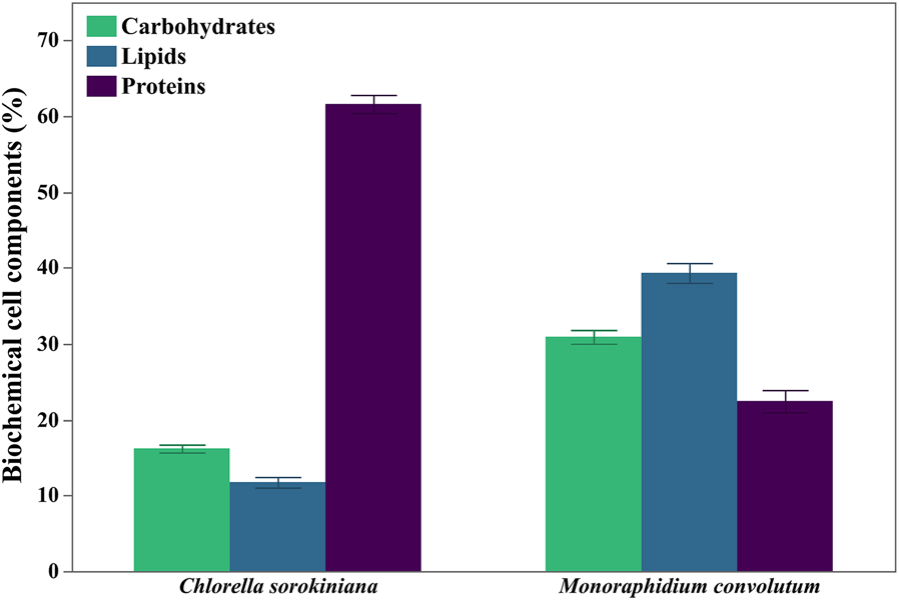
Biochemical composition of *Chlorella sorokiniana* and *Monoraphidium convolutum*, showing carbohydrate, lipid, and protein contents. Data is presented as means ± SE% (n = 3)

Figure 8 illustrates the biochemical composition of *C. sorokiniana* and *M. convolutum* grown on BBM. Concerning total carbohydrates and lipid contents, *C. sorokiniana* demonstrated lower contents (16.20 and 11.75%) compared to *M. convolutum* (30.94 and 39.31%), respectively. However, *C. sorokiniana* exhibited a significantly higher protein content (61.60%) in comparison with *M. convolutum* (22.46%).

### Sedimentation efficiency investigation

#### Monitoring sedimentation efficiency over time

**Fig. 9:**
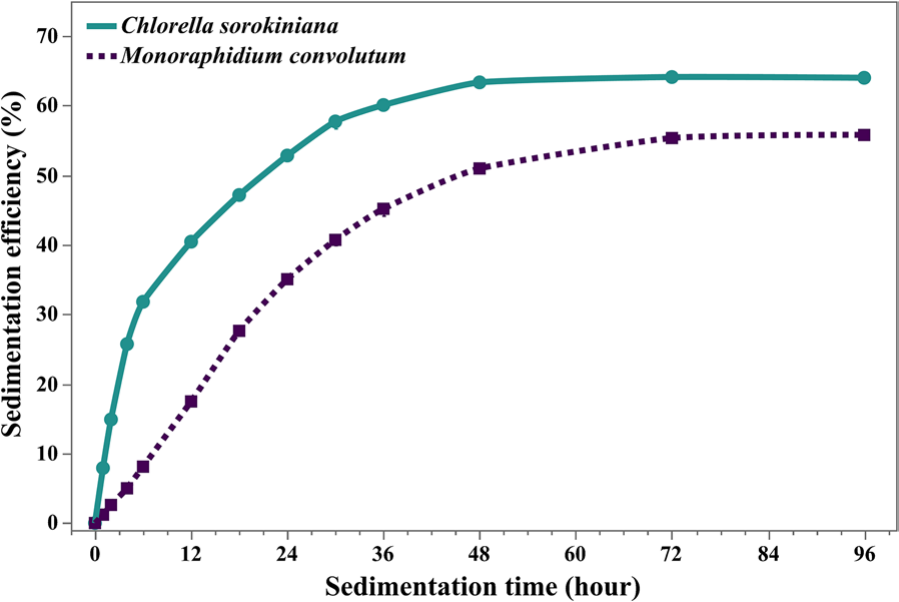
Temporal Analysis showing the effect of time on sedimentation efficiency for both species. Data is presented as means ± SE% (n = 3)

The sedimentation experiments revealed that *C. sorokiniana* exhibited a faster and more efficient sedimentation rate compared to *M. convolutum. C. sorokiniana* reached a sedimentation efficiency of 52.84% by the end of the first day, and a maximum significant value of 63.36% at 48 h. On the other hand, *M. convolutum* achieved only 35.06% for the first 24 h, 50.98% for the 2nd day and managed to reach the significant maximum sedimentation efficiency of 55.79% at 72 h as shown in Fig. 9.

#### Assessing the effect of gas exchange on sedimentation

**Fig. 10:**
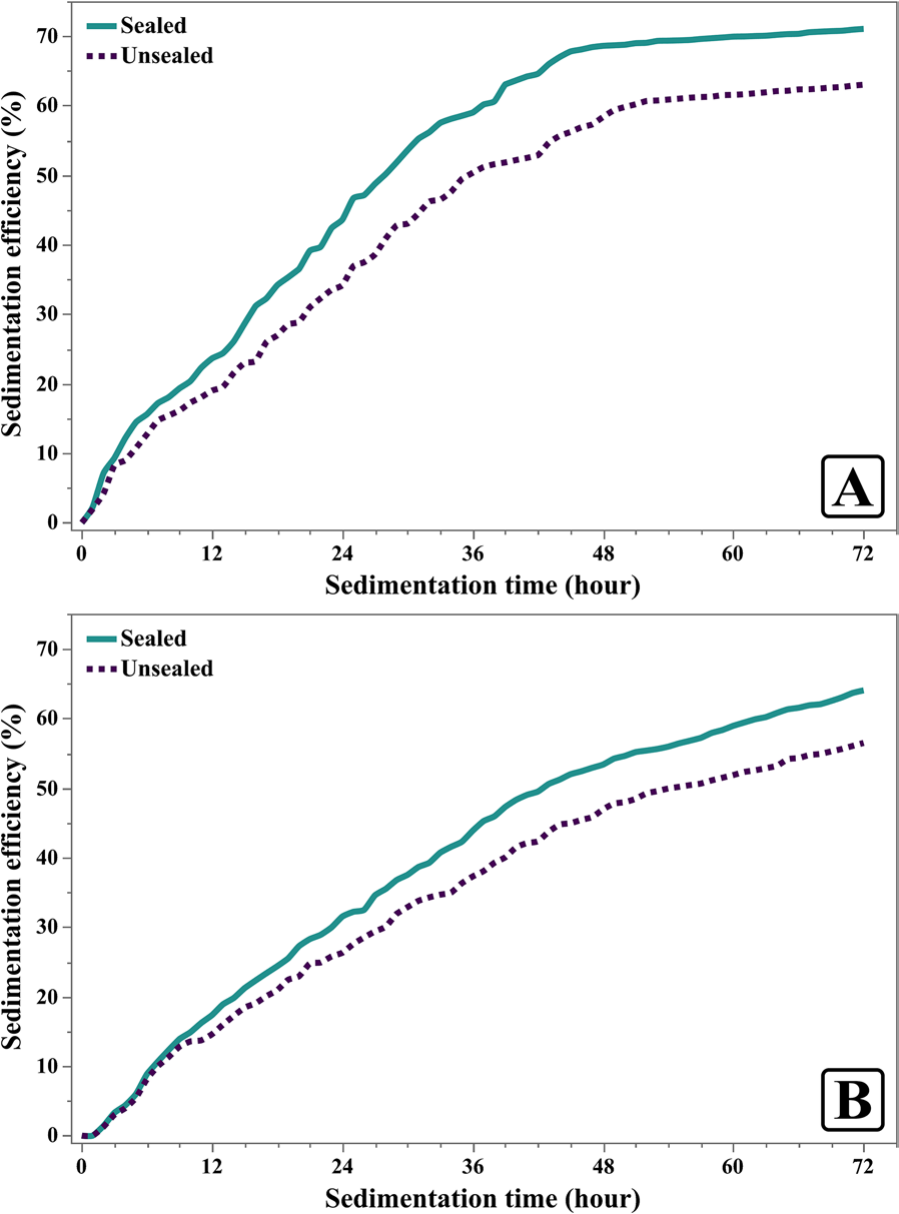
Line charts illustrating sedimentation efficiency (%) of *Chlorella sorokiniana* (**A**) and *Monoraphidium convolutum* (**B**) under sealed and unsealed conditions. Data is presented as means ± SE% (n = 3)

Data shown in Fig. 10 consolidate the significance of gas exchange on sedimentation efficiency where use of the sealed air-tight cylinders, which restricted gas exchange, resulted in higher sedimentation efficiencies for both *C. sorokiniana* and *M. convolutum C. sorokiniana* achieved 71.05% efficiency under sealed conditions compared to 63.01% in unsealed conditions (Fig. 10A) while reducing the necessary sedimentation time by approximately 5 h, while *M. convolutum* showed efficiencies of 64.06% sealed versus 56.5% unsealed (Fig. 10B) with no significant reduction in time.

#### Volume-based sedimentation efficiency analysis

**Fig. 11:**
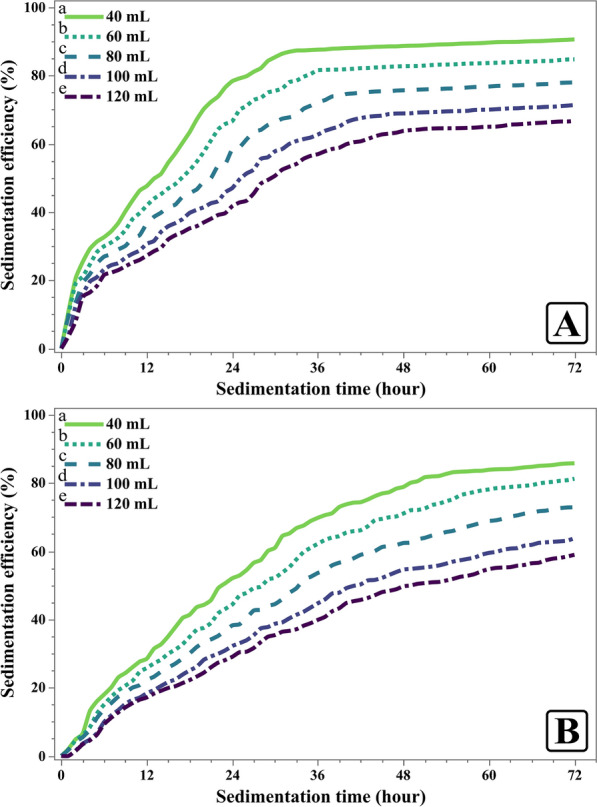
Effect of varying culture volumes on the sedimentation efficiency (%) of *Chlorella sorokiniana* (**A**) and *Monoraphidium convolutum* (**B**). Data is presented as means ± SE% (n = 3). Letters report on legend indicate significant differences at 72 h

The effect of culture volume on sedimentation efficiency was investigated, revealing a clear inverse pattern of response where lower volumes resulted in higher sedimentation efficiency (Fig. 11). For *C. sorokiniana*, 40 mL volume achieved 90.59% sedimentation efficiency along with 14.5 h reduction in time required for sedimentation, whereas 120 mL volume reached 66.68% only (Fig. 11A). Also, *M. convolutum* showed higher efficiency at lower volumes, where 40 mL volume reached 85.77% with about 12.25 h reduction compared to 58.95% sedimentation efficiency for 120 mL (Fig. 11B).

#### Sedimentation efficiency at different pH levels

**Fig. 12:**
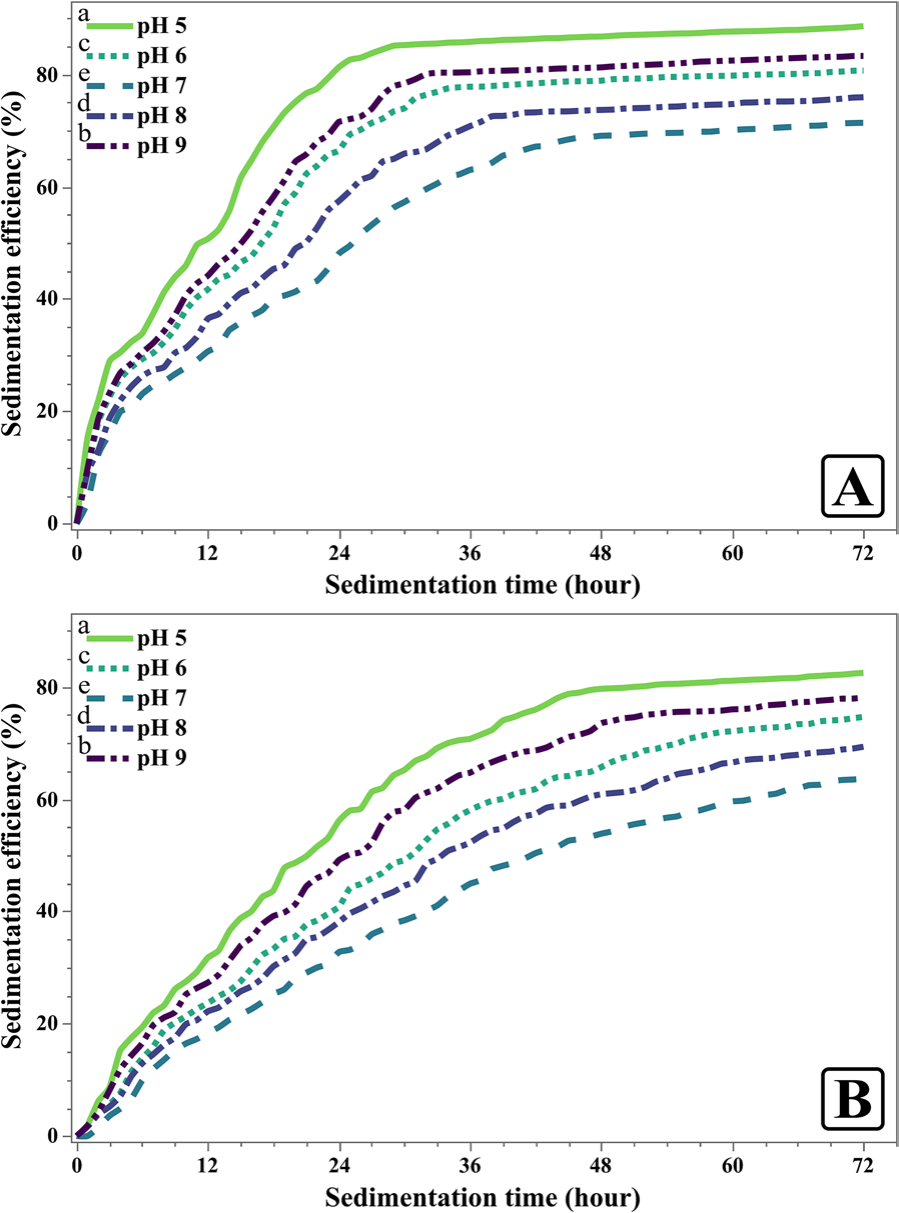
Sedimentation efficiency of *Chlorella sorokiniana* (**A**) and *Monoraphidium convolutum* (**B**) at various pH levels. Data is presented as means ± SE (n = 3). Letters report on legend indicate significant differences at 72 h while *p* ≤ 0.05

Data presented in Fig. 12 demonstrates how different levels of pH during the sedimentation process influence the sedimentation patterns for the two microalgae species. The sedimentation efficiency of *C. sorokiniana* varies with changes in pH (Fig. 12A). At pH 5, it exhibited the highest sedimentation efficiency of 88.60% in 30.75 h. As the pH increased to 6, efficiency decreased to 80.67% in 37.58 h. Sedimentation pH value of 7 exhibited a further decline in efficiency to 71.38% in 49.42 h. The efficiency improved slightly at pH 8, reaching 75.93% in 43.08 h, before rising again to 83.29% at pH 9 in 35.83 h.

For *M. convolutum* (Fig. 12B), the sedimentation efficiency followed a similar trend but at lower values. At pH 5, it achieved a sedimentation efficiency of 82.49% in 50.25 h. This value dropped at pH 6 to 74.64% in 63.42 h, and continued to decrease at pH 7, reaching 63.63% in 71.58 h. The efficiency improved at pH 8, reaching 69.34% in 64.83 h, before rising slightly again at pH 9 to 78.09% in 55.17 h.

#### Sedimentation efficiency under varying salinity levels

**Fig. 13:**
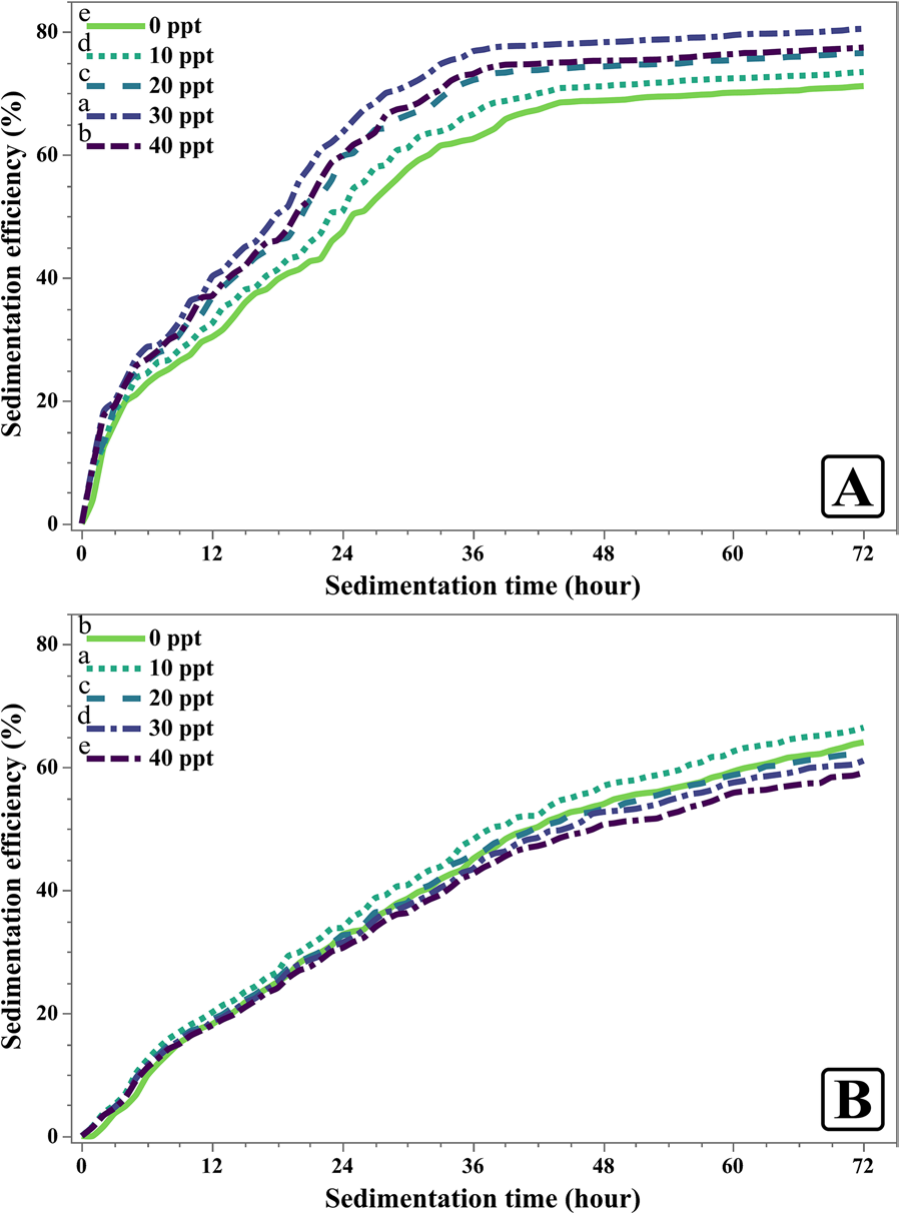
Sedimentation efficiency of *Chlorella sorokiniana* and *Monoraphidium convolutum* in different salinities by added NaCl (ppt). Data is presented as means ± SE (n = 3). Letters on legend indicate significant differences at 72 h

In Fig. 13, the sedimentation efficiency of *C. sorokiniana* increased progressively with rising salinity levels. At 0 ppt, *C. sorokiniana* exhibited a sedimentation efficiency of 71.22%, which improved all the way to 80.54% at 30 ppt along with a significant reduction of almost 10 h in the required time for sedimentation. But at 40 ppt, sedimentation efficiency was reduced to 77.49% and the required time went back up by 2 h.

In contrast, *M. convolutum* displayed a less consistent response to increasing salinity. At 0 ppt, the percentage of sedimentation efficiency was 64.10%. This value increased to 66.48% at 10 ppt but then declined to 62.19% at 20 ppt. The efficiency continued to decline till it reached 59.14% at 40 ppt with no significant reduction in the time of sedimentation.

### Enhancement of sedimentation responses using response surface methodology

**Fig. 14:**
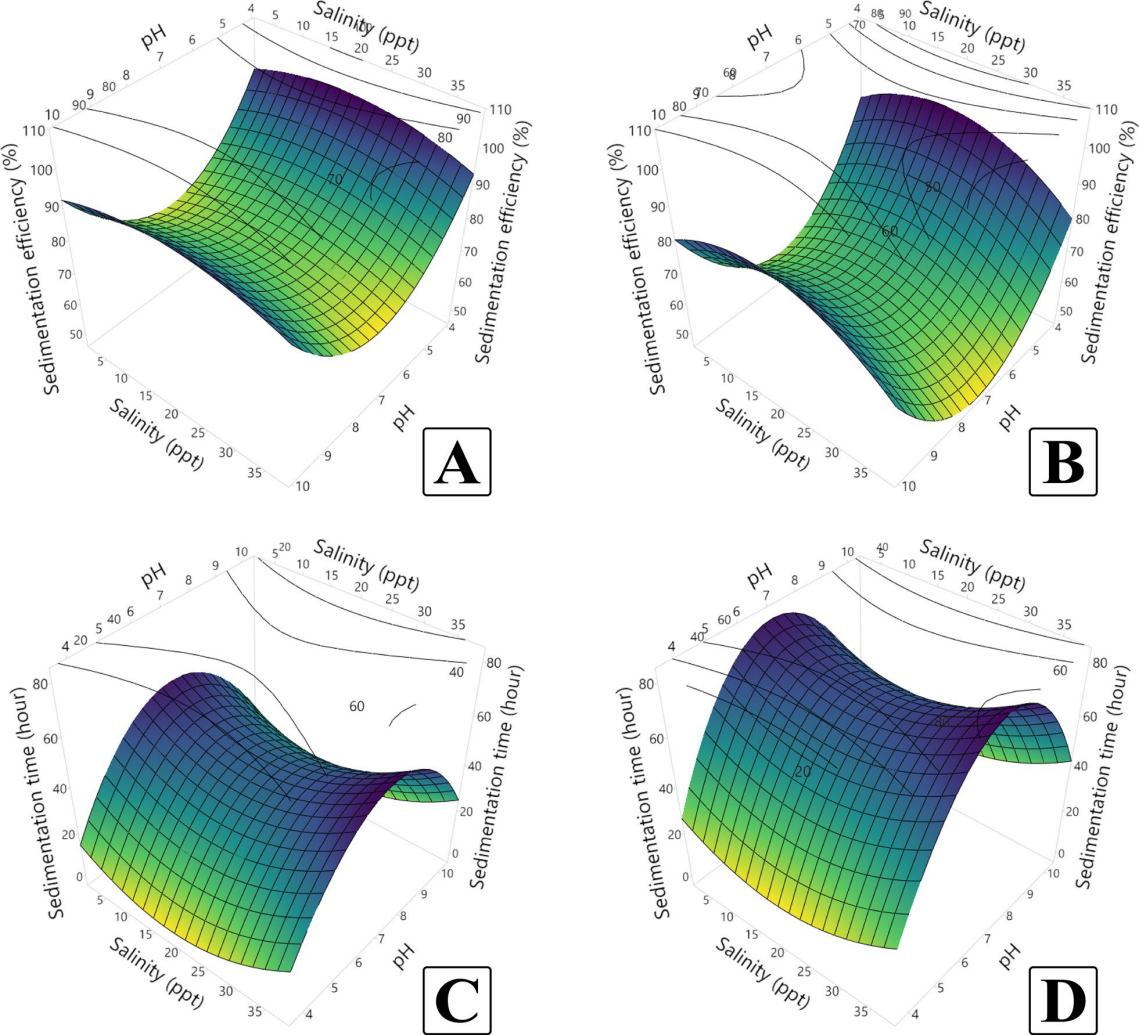
Three-dimensional response surface methodology (RSM) plots illustrating the effects of pH and salinity on sedimentation efficiency (*top*) and sedimentation time (*bottom*) for *Chlorella sorokiniana* (**A** and **C**, respectively) and *Monoraphidium convolutum* (**B** and **D**, respectively)

To study the effects of pH and salinity on sedimentation efficiency and time, a Central Composite Design (CCD) was employed for the two microalgae species, *C. sorokiniana* and *M. convolutum*. The experimental design included 14 runs divided into two blocks for practical applicability, with the independent variables (pH and salinity) assessed using a 5-level full factorial design as shown in Table 1. Experimental results are presented in Table 2 and Fig. 14.

**Table 2:** Central Composite Design (CCD) matrix with block distribution of the two studied input factors (pH and Salinity in ppt) with the output response values for sedimentation efficiency (%) and sedimentation time (hour) for each tested microalga

Run order	Blocks	pH	Salinity (ppt)	*Chlorella sorokiniana*		*Monoraphidium convolutum*	
				Efficiency (%)	Time (h)	Efficiency (%)	Time (h)
1	1	10	5	92.35	24.92	81.86	42.92
2	1	4	5	96.14	19.5	88.7	35.42
3	1	4	35	93.38	25.08	82.88	39.25
4	1	7	20	75.46	42.75	62.38	68.83
5	1	7	20	75.42	42.75	62.68	68.83
6	1	10	35	90.57	28.42	75.3	47.83
7	1	7	20	75.43	42.67	62.6	68.75
8	2	7	20	75.42	42.75	62.67	68.75
9	2	7	12.5	74.75	44.75	63.16	69.33
10	2	7	20	75.51	42.83	62.39	68.92
11	2	5.5	20	82.42	33.5	72.01	55.5
12	2	7	27.5	75.3	44.17	59.92	70.25
13	2	7	20	75.56	42.75	62.67	68.58
14	2	8.5	20	80.05	36.5	67.99	61.75

For *C. sorokiniana*, sedimentation efficiency ranged from 74.75 to 96.14%, with the highest efficiency observed at pH 4 and salinity 5 ppt (Run 2). This combination also resulted in one of the shortest sedimentation times, 19.5 h. Conversely, the lowest efficiency (74.75%) was recorded at pH 7 and salinity 12.5 ppt (Run 9), corresponding to a sedimentation time of 44.75 h. The sedimentation time varied more substantially, with the time ranging between 19.5 and 44.75 h, reflecting the sensitivity of the sedimentation process to environmental factors.

In the case of *M. convolutum*, sedimentation efficiency was lower overall, ranging from 59.92 to 88.7%. The highest efficiency (88.7%) occurred at pH 4 and salinity 5 ppt (Run 2), similar to *C. sorokiniana*. However, the lowest efficiency (59.92%) was observed at pH 7 and salinity 27.5 ppt (Run 12), a setting that also resulted in a prolonged sedimentation time of 70.25 h. Sedimentation time for *M. convolutum* was generally higher than that for *C. sorokiniana*, with values spanning from 35.42 to 70.25 h, indicating the need for tighter control of environmental conditions to enhance performance.

The fitted second-order polynomial models demonstrated excellent predictive capabilities, as shown by the model summary statistics (Table 3). For *C. sorokiniana*, the model for sedimentation efficiency exhibited an adjusted R^2^ of 99.61%, and a predicted R^2^ of 93.45%, reflecting the model’s robustness and reliability. The sedimentation time model also performed well, with an adjusted R^2^ of 99.08%, and a predicted R^2^ of 85.02%.

**Table 3:** Model summary statistics for sedimentation response variables for each microalga

Microalgae	Response	S^a^	R^2b^	R^2^ (adj)^c^(%)	R^2^ (pred)^d^(%)
*Chlorella sorokiniana*	Efficiency (%)	0.508063	99.79%	99.61	93.45
	Time (h)	0.83676	99.50%	99.08	85.02
*Monoraphidium convolutum*	Efficiency (%)	0.39806	99.90%	99.82	99.38
	Time (h)	0.850932	99.76%	99.56	94.58

^a^Standard error of the regression

^b^Percentage of variation in the response

^c^Adjusted percentage of variation relative to the number of observations

^d^Indicator of model prediction accuracy

For *M. convolutum*, the models displayed even stronger predictive performance. The sedimentation efficiency model achieved an adjusted R^2^ of 99.82%, and a predicted R^2^ of 99.38%, indicating near-perfect accuracy. Similarly, the sedimentation time model exhibited an adjusted R^2^ of 99.56%, and a predicted R^2^ of 94.58%.

Analysis of variance (ANOVA) in Tables 4 and 5 further highlighted the importance of pH and salinity in influencing sedimentation efficiency and time. For *C. sorokiniana*, the linear terms for pH (*p*-value < 0.001) and salinity (*p*-value = 0.005) were statistically significant, as were the quadratic terms (pH^2^ *p*-value < 0.001, Salinity^2^ *p*-value = 0.011). The interaction term (pH*Salinity) was not significant (*p*-value = 0.367). Similar trends were observed for *M. convolutum*, where pH and salinity showed significant linear and quadratic effects on efficiency (*p*-value < 0.001 for all), while the interaction term remained insignificant (*p*-value = 0.384).

**Table 4:** Analysis of variance (ANOVA) and effect tests of the input factors (pH and salinity) of the response surface CCD model for sedimentation responses (efficiency and time) in *Chlorella sorokiniana*

Source	DF	Efficiency (%)				Time (h)			
		Adj SS^a^	Adj MS^b^	F-value^c^	*p*-value^d^	Adj SS	Adj MS	F-value	*p*-value
Model	6	858.269	143.045	554.16	<0.001*	983.786	163.964	234.18	<0.001*
Blocks	1	0.251	0.251	0.97	0.357	0.434	0.434	0.62	0.457
Linear	2	17.51	8.755	33.92	<0.001*	40.563	20.281	28.97	<0.001*
pH	1	13.468	13.468	52.18	<0.001*	23.393	23.393	33.41	0.001*
Salinity	1	4.042	4.042	15.66	0.005*	17.17	17.17	24.52	0.002*
Square	2	585.6	292.8	1134.32	<0.001*	674.96	337.48	482	<0.001*
pH*pH	1	109.315	109.315	423.49	<0.001*	190.41	190.41	271.95	<0.001*
Salinity*Salinity	1	3.054	3.054	11.83	0.011*	22.948	22.948	32.77	0.001*
2-way interaction	1	0.24	0.24	0.93	0.367	1.082	1.082	1.54	0.254
pH*Salinity	1	0.24	0.24	0.93	0.367	1.082	1.082	1.54	0.254
Error	7	1.807	0.258			4.901	0.7		
Lack-of-fit	3	1.796	0.599	219.02	<0.001*	4.893	1.631	764.47	<0.001*
Pure error	4	0.011	0.003			0.009	0.002		
Total	13	860.076				988.687			

^a^Adjusted sum of squares

^b^Adjusted mean squares

^c^Fishers’s exact test statistic value

^d^Probability of significance against the null hypothesis

^*^Significant at 90% level of confidence (*p*-value < 0.1)

**Table 5:** Analysis of variance (ANOVA) and effect tests of the input factors (pH and salinity) of the response surface CCD model for sedimentation responses (efficiency and time) in *Monoraphidium convolutum*

Source	DF	Efficiency (%)				Time (h)			
		Adj SS^a^	Adj MS^b^	F-value^c^	*p*-value^d^	Adj SS	Adj MS	F-value	*p*-value
Model	6	1159.79	193.298	1219.92	<0.001*	2145.32	357.554	493.8	<0.001*
Blocks	1	0.56	0.562	3.55	0.102	1.33	1.334	1.84	0.217
Linear	2	103.54	51.772	326.73	<0.001*	100.77	50.386	69.59	<0.001*
pH	1	59.99	59.988	378.59	<0.001*	81.96	81.963	113.19	<0.001*
Salinity	1	43.56	43.556	274.88	<0.001*	18.81	18.809	25.98	0.001*
Square	2	748.82	374.41	2362.93	<0.001*	1449.51	724.753	1000.9	<0.001*
pH*pH	1	173.1	173.096	1092.42	<0.001*	314.91	314.906	434.9	<0.001*
Salinity*Salinity	1	12.02	12.025	75.89	<0.001*	17.59	17.59	24.29	0.002*
2-Way interaction	1	0.14	0.137	0.86	0.384	0.29	0.292	0.4	0.546
pH*Salinity	1	0.14	0.137	0.86	0.384	0.29	0.292	0.4	0.546
Error	7	1.11	0.158			5.07	0.724		
Lack-of-fit	3	1.01	0.336	13.38	0.015*	5.01	1.669	107.55	<0.001*
Pure error	4	0.1	0.025			0.06	0.016		
Total	13	1160.9				2150.39			

^a^Adjusted sum of squares

^b^Adjusted mean squares

^c^Fishers’s exact test statistic value

^d^Probability of significance against the null hypothesis

^*^Significant at 90% level of confidence (*p*-value < 0.1)

The derived regression equations capture these relationships and offer insights into the trends observed, as shown in the second-order polynomial equations for *C. sorokiniana* (Eqs. 6 and 7):

6 $$Efficiency\ \left(\%\right)= 191.18 - 33.79\ pH + 0.531\ Salinity + 2.365\ {pH}^{2} - 0.01581\ {Salinity}^{2} + 0.00544\ pH*Salinity$$

7 $$Time\ \left(Hours\right)= -102.57 + 44.68\ pH - 1.522\ Salinity - 3.121\ {pH}^{2} + 0.04334\ {Salinity}^{2} - 0.01156\ pH*Salinity$$

And the following equations for *M. convolutum* (Eqs. 8 and 9):

8 $$Efficiency\ (\%) = 208.10 - 42.79\ pH + 1.076\ Salinity + 2.9757\ {pH}^{2} - 0.03137\ {Salinity}^{2} - 0.00411\ pH*Salinity$$

9 $$Time\ (Hours) = -124.83 + 57.49\ pH - 1.423\ Salinity - 4.014\ {pH}^{2} + 0.03794\ {Salinity}^{2} + 0.00600\ pH*Salinity$$

Validation of the optimized conditions (Table 6) confirmed the reliability of the models. For *C. sorokiniana*, the predicted sedimentation efficiency of 98.68% and time of 12.06 h at pH 4 and salinity 17.4 ppt were close to the actual values of 95.89% and 18.75 h. For *M. convolutum*, predictions of 93.48% efficiency and 28.12 h at pH 4 and salinity 16.8 ppt aligned closely with observed values of 89.2% and 33.91 h, respectively.

**Table 6:** The actual and predicted values for sedimentation efficiency and time at input settings derived from the optimized response surface CCD model for each microalga

Microalgae	Setting		Predicted response		Actual response	
	pH	Salinity	Efficiency (%)	Time (h)	Efficiency (%)	Time (h)
*Chlorella sorokiniana*	4	17.4	98.68	12.06	95.89	18.75
*Monoraphidium convolutum*	4	16.8	93.483	28.12	89.2	33.91

### Economic cost reduction estimates

To compare the economic feasibility of harvesting methods, the cost per kilogram of dry biomass using sedimentation was evaluated versus centrifugation. Centrifugation, with an average energy demand of 5.8 kWh m^−3^ and an energy rate of $0.33/kWh, incurs a cost of approximately $1.914 per m^3^. In contrast, the optimized sedimentation protocol in CCD run no. 2 (pH 4, 5 ppt NaCl) requires only 5 kg of NaCl and about 10 mL of commercial 33% HCl per m^3^, totaling $0.42761 per m^3^.

Using experimental biomass yields and recovery efficiencies, the total cost to harvest 1 kg of biomass was estimated for both species and methods. As shown in Table 7, sedimentation reduced harvesting costs by up to 79% for *C. sorokiniana* and up to 77% for *M. convolutum* compared to centrifugation, while achieving comparable recovery.

**Table 7:** Comparative analysis of harvesting efficiency, biomass yield, required culture volume, and total harvesting cost per kilogram of biomass for each species using optimized sedimentation and conventional centrifugation

Parameter	*Chlorella sorokiniana*		*Monoraphidium convolutum*	
	Sedimentation	Centrifugation	Sedimentation	Centrifugation
Harvesting efficiency (%)	96.14	90	88.7	90
Harvested biomass (mg L^−1^)	422.37	395.4	604.93	613.8
Volume per kg biomass (m^3^ kg^−1^)	2.368	2.529	1.653	1.629
Harvesting cost ($USD kg^−1^)	1.012	4.841	0.707	3.118

### Validation of non-invasive photographic method for sedimentation efficiency

**Fig. 15:**
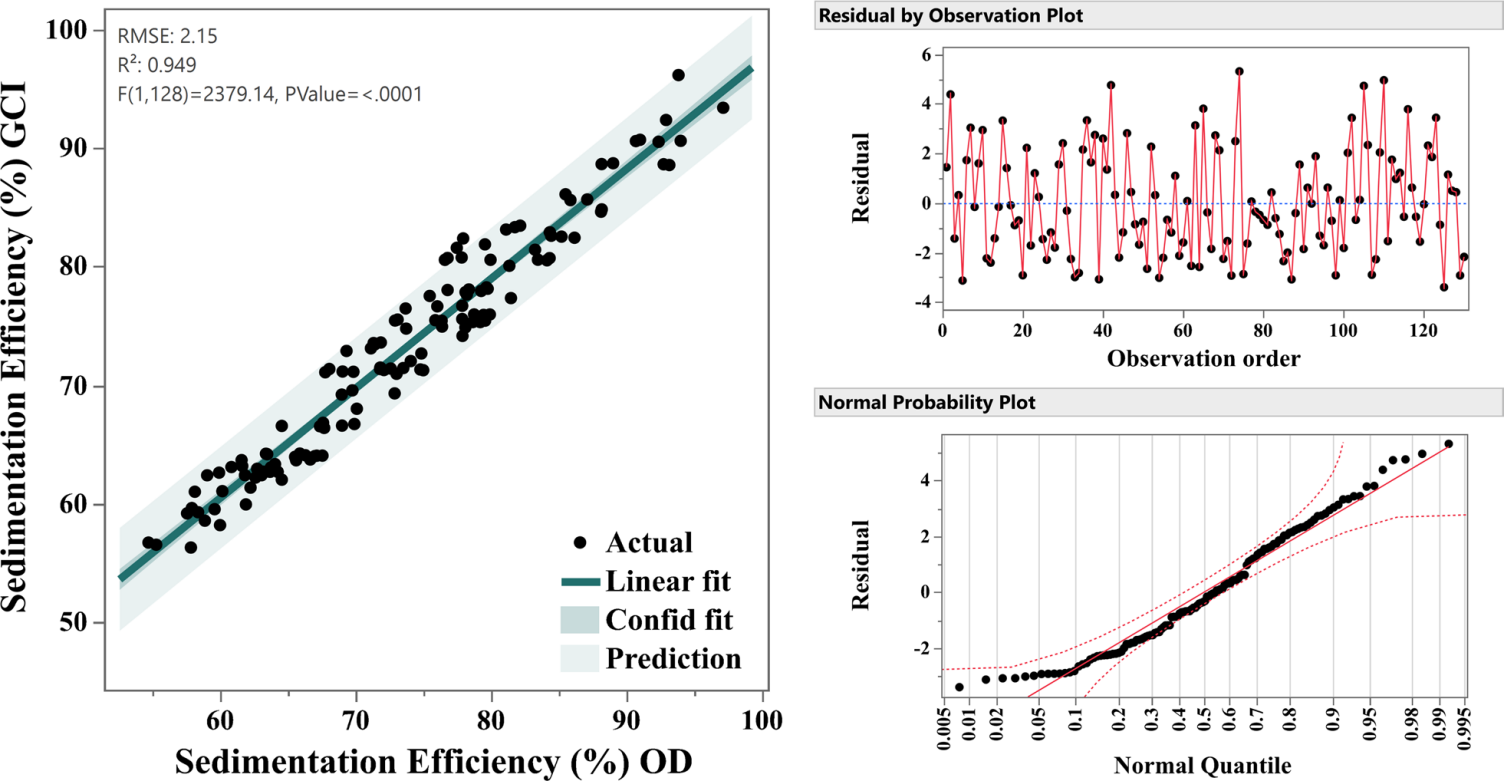
Comparative validation between SE_GCI_ and SE_OD_ techniques using linear regression analysis (*left*), Residual analysis (*top-right*) signifies the general consistency of fit and normal probability plot (*bottom-right*) shows residuals distribution all highlighting the high accuracy of the method

The non-invasive photographic method developed in this study was validated by comparing sedimentation efficiency measured through optical density (SE_OD_) with sedimentation efficiency measured through the green color intensity (SE_GCI_). A regression analysis was performed to assess the relationship between the two methods (Fig. 15).

The model summary showed an R^2^ value of 94.89%, indicating that the photographic method experienced a proportion of variance in the sedimentation efficiency measured. The adjusted R^2^ value of 94.85% and the predicted R^2^ of 94.75% confirmed the robustness and predictive power of the model. The Pearson coefficient (*r*) between both methods (0.93) was statistically significant (*p*-value < 0.001), indicating a very strong positive linear relationship which further proves that the non-invasive (SE_GCI_) automated method is highly reliable and efficient.

### Correlation and regression estimation between sedimentation factors and responses

**Fig. 16:**
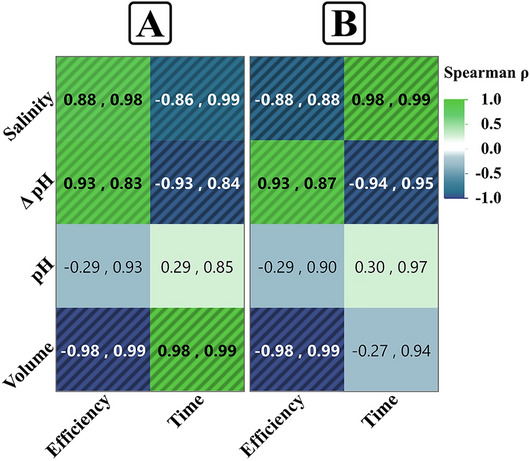
Correlation heatmap for the relationship between experimental parameters (salinity, pH, ΔpH, and volume) and sedimentation outcomes (efficiency and time). Data represent correlation and regression (Spearman's *ρ*, adjusted R^2^) for *Chlorella sorokiniana* (**A**) and *Monoraphidium convolutum* (**B**). The shaded stripes ▨ highlight statistically significant correlations (*p*-value < 0.05)

To understand the relationships between the factors tested, pH, salinity, volume, and ΔpH (absolute change from neutral pH), and the responses measured (sedimentation efficiency and sedimentation time), Spearman’s correlation (*ρ*) and regression analysis were applied, and the obtained results are shown in Fig. 16. This analysis revealed the extent of association and the predictive strength of the relationships across the microalgal species *C. sorokiniana* and *M. convolutum*.

The correlation analysis highlighted varying trends between the factors and sedimentation efficiency. For pH, a non-significant weak negative correlation (*ρ* = −0.29) was observed for both species, indicating minimal influence of pH changes on efficiency. Conversely, salinity displayed contrasting correlations. For *C. sorokiniana*, salinity was significantly strong and positively correlated with efficiency (*ρ* = 0.88), while for *M. convolutum*, the relationship was strongly negative (*ρ* = −0.88). Volume consistently exhibited a significant strong negative correlation with efficiency for both species (*ρ* = −0.98), suggesting that smaller volumes increase sedimentation efficiency. Interestingly, ΔpH had a significant strong positive correlation with efficiency (*ρ* = 0.93), emphasizing that deviations from neutral pH significantly improve sedimentation outcomes.

When examining correlations with sedimentation time, pH demonstrated a weak non-significant positive association (*ρ* = 0.29) for both microalgae, suggesting a limited role in predicting sedimentation time. Salinity showed inverse relationships across species: a significant strong negative correlation for *C. sorokiniana* (*ρ* = −0.86) and a strong positive correlation for *M. convolutum* (*ρ* = 0.98). Volume also exhibited species-dependent trends, with a strong significant positive correlation for *C. sorokiniana* (*ρ* = 0.98) but a weak non-significant negative correlation for *M. convolutum* (*ρ* = −0.27). ΔpH revealed a strong negative correlation with sedimentation time (*ρ* = −0.93 for *C. sorokiniana* and *ρ* = −0.94 for *M. convolutum*), demonstrating that greater deviations from neutral pH reduce the time required for sedimentation.

The regression analysis revealed that most interactions between the factors (pH, salinity, volume, and ΔpH) and the responses (sedimentation efficiency and sedimentation time) resulted in high adjusted R^2^ values, demonstrating strong predictive relationships. However, certain factors showed weaker predictive power. pH demonstrated relatively lower adjusted R^2^ values for sedimentation efficiency (R^2^ adj = 0.9287 for *C. sorokiniana* and R^2^ adj = 0.9048 for *M. convolutum*), while ΔpH, despite its strong correlations, resulted in moderate adjusted R^2^ values (R^2^ adj = 0.8318 for *C. sorokiniana* and R^2^ adj = 0.8721 for *M. convolutum*).

For sedimentation time, similar trends were observed. Both salinity and volume produced extremely high adjusted R^2^ values, such as 0.9964 and 0.9988, respectively, for *C. sorokiniana*. ΔpH also exhibited a strong fit, particularly for *M. convolutum* (R^2^ adj = 0.9465). On the other hand, pH displayed the weakest predictive capability among the factors, with adjusted R^2^ adj values of 0.8484 for *C. sorokiniana* and 0.9727 for *M. convolutum*.

## Discussion

The findings from this study reveal important insights into the growth and sedimentation behaviors of *C. sorokiniana* and *M. convolutum* under various conditions. These results are essential for optimizing microalgae harvesting, which particularly has many significant applications such as biofuel production, wastewater treatment, and biochemicals production.

The growth performance of *C. sorokiniana* and *M. convolutum* assessment revealed that Bold Basal Medium consistently supported the maximum levels of growth across all metrics, which indicated a vigorous growth phase, characterized by rapid cell proliferation and biomass accumulation. The present data is supported by the previous findings of Guerrero-Cabrera et al. [28], who studied the growth of three different microalgae, *Monoraphidium* sp., *Chlorella* sp. and *Scenedesmus* sp., in BBM compared with Nile Tilapia Effluent Medium and suggested that the usage of BBM generally resulted in higher positive responses concerning growth rate, biomass, as well as lipid and protein contents. Moreover, Da Silva et al. [54] investigated the physiologic parameters of *C. sorokiniana* CTT7727 at different growth conditions on BBM and concluded that BBM with continuous illumination and CO_2_ feeding resulted in maximum cell concentration and growth rate in *C. sorokiniana*, emphasizing the importance of balanced nutrients in promoting vigorous growth in microalgae. The correlating harmony observed between results of culture density, cell count, and biomass in the present study aligns with these findings, reinforcing the role of nutrient-rich media like BBM in promoting algal growth. This can also be further proved by BBM’s ability to support a higher specific growth rate generally (0.31 day^−1^ for *C. sorokiniana* and 0.34 day^−1^ for *M. convolutum*) which is also noticeable by its considerably short lag phase along with higher growth rates during the first 4 days (0.49 day^−1^ for both species) as well as the later exponential phase (Days 4–8) specially in the case of *M. convolutum*. This is supported by Okomoda et al. [55], whose findings on *Ankistrodesmus falcatus* growth habits in BBM, Modified COMBO, and Bristol medium recognized that BBM showed higher biomass production and specific growth rates compared to Bristol medium. However, Dayananda et al. [56] found that BG11 can be more suitable for hydrocarbon and biomass production in *Botryococcus braunii*, a freshwater microalgae, as deduced by their work using different autotrophic media as BBM, BBMa (with ammonium carbonate), BG11, and modified Chu 13. Additionally, Asma et al. [57] concluded that natural media can outperform chemically formulated media for some species as *B. braunii* had a higher specific growth rate in autoclaved lake water compared to its moderate growth in BBM and modified Chu No. 10 medium. This discrepancy may be attributed to differences in species-specific nutrient requirements. Present data of *M. convolutum* grown in BBM illustrated higher dry weight content than that of *C. sorokiniana*, despite the latter having higher culture density which can be attributed to the higher specific growth rate across the different intervals documented in this study and also the accumulation of higher lipid and carbohydrate contents in *M. convolutum* compared to that of *C. sorokiniana*. This observation aligns with the findings of Díaz et al. [8] and Matsukawa et al. [7], who suggested that *Monoraphidium* species are capable of higher lipids (30.58%) and carbohydrates (25.58%) accumulation while *C. sorokiniana* has higher protein content (68.5%).

In the current study, the faster sedimentation rate of *C. sorokiniana* may be attributed to cell morphology and biochemical composition. Whereas reduced buoyancy of *C. sorokiniana*, due to its smaller cell size and lower oil content, facilitates a shorter settling time. This observation aligns with the findings of Baroni et al. [58], who also linked cell size and cell density with the time of gravity sedimentation in microalgae (*Chlorella* sp., *Haematococcus pluvialis*, and *Nannochloropsis salina*). Furthermore, in their study, nitrogen depletion increased the average cell size by 30% and decreased cell density, which in turn reduced the settling velocity. The increase in cell size only partially counteracts the decrease in density, leading to a slower sedimentation rate. Also, Sung et al. [59], who used a sedimentation rate-based strain selection to study microalgae for use as direct combustion fuels with lower CO_2_ emissions, concluded that high-lipid content strains settle more slowly due to increased buoyancy. This confirms the findings of this study as *C. sorokiniana* possesses smaller spherical cells with higher density, due to the higher protein content, in comparison with *M. convolutum*, with its higher oil content and larger cells with ellipsoid shape. All resulting in a lower time of sedimentation and higher efficiency in *C. sorokiniana* compared to *M. convolutum*.

The current data suggested that limiting gas exchange reduces metabolic activity and alters buoyancy, leading to higher sedimentation rates. This finding is supported by studies of Tamburic et al. [60], who demonstrated the role of CO_2_ availability in microalgal metabolism and its subsequent effect on sedimentation rates as their study directly linked carbon limitation to reduced metabolic rates in *Nannochloropsis oculata*, showing that gas exchange governs the extent of carbon limitation which could lead to higher sedimentation due to reduced metabolic activity. Also, Cheng et al. [61] demonstrated how microalgae like *Chlorella vulgaris* cultured in closed systems can experience altered gas exchange, where CO_2_ is removed, affecting cellular processes. The results demonstrate that low gas exchange slows CO_2_ fixation and oxygen production, affecting cell growth and metabolism. These results suggest that controlling gas exchange could be a viable strategy for improving sedimentation efficiency as a feasible method in large-scale microalgal cultivation.

The sedimentation efficiency of *C. sorokiniana* and *M. convolutum* was significantly influenced by culture volume, with lower culture volumes consistently demonstrating higher sedimentation efficiencies for both species. This decline in efficiency with increasing culture volume can be attributed to the greater height of the culture column, which results in increased fluid resistance and enhanced buoyancy forces acting on the algal cells. Relational analysis confirmed a strong negative correlation between culture volume and sedimentation efficiency for both species (*ρ* = −0.98, *p*-value < 0.001), indicating a predictable decrease in efficiency as culture volume increases. The regression analysis further supported the reliability of this relationship, with adjusted R^2^ values of 0.99 for both *C. sorokiniana* and *M. convolutum*. In addition, sedimentation time exhibited a positive correlation with culture volume in *C. sorokiniana* (*ρ* = 0.98, *p*-value < 0.001, R^2^ = 0.99), reflecting prolonged settling times in larger culture volumes. Conversely, *M. convolutum* displayed a weaker and statistically insignificant correlation between culture volume and sedimentation time (*ρ* = −0.27, *p*-value = 0.32), suggesting that its buoyancy characteristics, likely influenced by higher oil content, make it less sensitive to changes in volume when considering settling time. These findings emphasize the importance of optimizing culture volume in sedimentation processes. Smaller volumes effectively mitigate fluid resistance and buoyancy effects, resulting in improved sedimentation efficiency. This aligns with the observations of Hirom and Devi [62], who reported that larger tanks are associated with issues like increased fluid resistance, which negatively impacts the settling rate of suspended particles. The present study reinforces these conclusions, providing additional evidence that sedimentation efficiency can be maximized by reducing culture volume, especially for species with distinct biochemical compositions, such as *M. convolutum*.

The sedimentation efficiency of *C. sorokiniana* and *M. convolutum* exhibited a non-linear relationship with pH, with the highest efficiencies observed under acidic (pH 5) and alkaline (pH 9) conditions, while efficiencies were lower at neutral pH (pH 7). For *C. sorokiniana*, sedimentation efficiency peaked at 88.60% at pH 5 and 83.29% at pH 9, compared to 71.38% at pH 7. Similarly, for *M. convolutum*, efficiencies were 82.49% at pH 5 and 78.09% at pH 9, significantly higher than the 63.63% observed at neutral pH. This trend can be explained by the effect of pH on the surface charge of algal cells, which in turn promotes cellular aggregation and enhances sedimentation. Acidic conditions may neutralize negative surface charges on the cell membrane, reducing electrostatic repulsion, as previously noted by Castrillo et al. and Tan et al. [63, 64]. Alkaline conditions, on the other hand, have been associated with auto-flocculation mechanisms, as demonstrated by Wu et al. [65], who reported enhanced settling rates of up to 90% in freshwater microalgae at elevated pH values. Regression and correlation analyses further highlight the influence of pH and ΔpH. The relationship between pH and sedimentation efficiency showed weak negative correlations (*ρ* = −0.29, *p*-value = 0.286) for both species. However, when analyzed as ΔpH (the absolute change from neutral pH), a strong positive correlation emerged (*ρ* = 0.93, *p*-value < 0.0001), confirming the significance of deviations from neutrality in driving sedimentation efficiency. The adjusted R^2^ values for ΔpH efficiency relationships were 0.83 for *C. sorokiniana* and 0.87 for *M. convolutum*, highlighting the robustness of these findings. Sedimentation times were inversely correlated with ΔpH for both species, with stronger relationships observed for *M. convolutum* (*ρ* = −0.94, *p*-value < 0.0001, R^2^ = 0.95). This suggests that larger deviations from neutral pH not only improve efficiency but also reduce settling time, especially in *M. convolutum*. These results align with prior studies on *Scenedesmus obliquus* and *Chlorella vulgaris* where pH values between 10 and 12 were used [63], and in *C. sorokiniana* at pH 12, achieving up to 97.8% sedimentation efficiency [66]. The cell wall of *C. sorokiniana* is known to be relatively rigid and protein-rich, contributing to its stability and compactness, which may enhance sedimentation under acidic or saline conditions [67, 68]. In contrast, *M. convolutum* possesses a thinner, more flexible cell wall, which, combined with higher lipid content, could lead to increased buoyancy and reduced responsiveness to ionic or pH-driven aggregation. The current findings reinforce the importance of optimizing pH conditions in sedimentation processes to maximize efficiency while minimizing adverse effects on cell integrity, potential stress on microalgal cells and biomass quality.

Salinity played a crucial role in modulating sedimentation efficiency, with contrasting effects observed between *C. sorokiniana* and *M. convolutum*. For *C. sorokiniana*, sedimentation efficiency increased significantly with rising salinity, reaching a maximum of 80.54% at 30 ppt but declined to 77.49% at 40 ppt. Regression analysis confirmed a strong positive correlation between salinity and sedimentation efficiency (*ρ* = 0.88, *p*-value < 0.001, R^2^ = 0.98), indicating a predictable enhancement in sedimentation efficiency with salinity stress. This trend was accompanied by a decrease in sedimentation time (*ρ* = −0.86, *p*-value < 0.001, R^2^ = 0.99), suggesting that sedimentation in *C. sorokiniana* can be promoted and accelerated with elevating salinity up to certain levels below hypersaline environments where it would have an adverse effect on the process. The observed improvement in sedimentation efficiency for *C. sorokiniana* can be attributed to physiological adaptations triggered by salinity stress. Previous studies, such as those by Kim et al. [69], have demonstrated that salinity stress induces lipid accumulation, increasing cell density and promoting faster settling rates. Furthermore, salinity stress may enhance the production of extracellular polymeric substances (EPS), which contribute to sediment biostabilization, as reported by Spears et al. [70] in benthic microalgae. These physiological changes likely explain the higher sedimentation efficiency observed at elevated salinity levels. In contrast, *M. convolutum* exhibited a varying response and decline in sedimentation efficiency with increasing salinity, with efficiency slightly increasing at low salinity (10 ppt), then dropping from 64.10 to 59.14% at 40 ppt. Regression analysis revealed a significant negative correlation between salinity and sedimentation efficiency (*ρ* = −0.88, *p*-value < 0.001, R^2^ = 0.88), highlighting the adverse effect of salinity on this species. Interestingly, sedimentation time for *M. convolutum* increased with salinity (*ρ* = 0.98, *p*-value < 0.001, R^2^ = 0.99), suggesting that higher salinity levels not only reduce efficiency but also prolong settling time. These results align with earlier findings by Díaz et al. [8] and Matsukawa et al. [7] supporting that *M. convolutum* may not exhibit similar physiological responses leading to limited response to salinity which can be attributed to its higher lipid content and lower halotolerance as indicated by Talebi et al. [71] whose work suggested that some microalgae species with lower salinity tolerance often show diminished responses in cellular alterations likely limiting their ability to undergo significant physiological alterations under salt stress.

The analysis of variance (ANOVA) and effect tests in the CCD response surface model revealed that both pH and salinity had significant effects on sedimentation efficiency and time for both microalgae species. For *C. sorokiniana*, the linear terms of pH (*p*-value < 0.001) and salinity (*p*-value = 0.005) were significant contributors to efficiency, with pH demonstrating a stronger impact based on its higher F-value. Similarly, salinity significantly influenced sedimentation time (*p*-value = 0.002). Quadratic terms (pH^2^ and Salinity^2^) also had a distinct effect, indicating that extreme values of these factors either reduced or plateaued out sedimentation performance. For *M. convolutum*, a comparable trend was observed, with pH emerging as the most influential factor for both efficiency (*p*-value < 0.001) and time (*p*-value < 0.001). The quadratic effects of pH (*p*-value < 0.001) further emphasized the sensitivity of this species to pH variations, reflecting a higher degree of optimization required for its flocculation. While salinity was also significant, its relative impact was lesser compared to pH. Interestingly, the 2-way interaction term (pH*Salinity) was not statistically significant (*p*-value > 0.01) for either species, suggesting that the two factors operate independently rather than synergistically to influence sedimentation. This independence simplifies the optimization process, allowing each factor to be fine-tuned separately. These findings are supported by Zalizniak et al. [72] and Grenier et al. [73], who confirmed that the changes in pH did not significantly modify the direct effects of salinity and that there was no significant combined effect of pH and salinity. However, results also showed that applying both pH and salinity within their respective optimal ranges led to enhanced sedimentation outcomes compared to either factor alone. This indicates that while synergy is absent, their combined influence remains practically beneficial and should be considered in applied harvesting strategies.

The predictive models for sedimentation efficiency and time demonstrated exceptional accuracy, as evidenced by high R^2^, adjusted R^2^, and predicted R^2^ values for both species. The corresponding predictive capabilities confirm the robustness of the models in forecasting outcomes under untested conditions. This highlights the superior adaptability of the model for these species, which may stem from more consistent biological responses under varying environmental conditions. Validation of the optimized settings confirmed the reliability of the models. For *C. sorokiniana*, the predicted sedimentation efficiency of 98.68% at pH 4 and salinity 17.4 ppt closely matched the actual efficiency of 95.89%. Similarly, sedimentation time predictions (12.06 h) underestimated the observed time (18.75 h) but remained within acceptable limits of accuracy. For *M. convolutum*, the predicted efficiency of 93.48% under optimal conditions (pH 4, salinity 16.8 ppt) was slightly higher than the actual efficiency (89.2%), while predicted and actual sedimentation times (28.12 vs. 33.91 h) exhibited minor discrepancies. These results reinforce the utility of the models in guiding process optimization, while also underscoring the inherent variability in biological systems.

The observed differences in sedimentation efficiency between *Chlorella sorokiniana* and *Monoraphidium convolutum* can be partially attributed to species-specific morphological and biochemical traits. *C. sorokiniana* exhibits small, spherical cells that are typically denser, whereas *M. convolutum* displays larger, ellipsoidal cells with a higher lipid content—characteristics that influence settling behavior through variations in buoyancy and surface area-to-volume ratios. Although this study did not include direct measurements of cell surface charge (zeta potential) or extracellular polymeric substances (EPS), both factors are known to play a critical role in microalgal aggregation and flocculation. Negatively charged cell surfaces may inhibit sedimentation unless neutralized by changes in pH or ionic strength [74], while EPS can either promote or hinder aggregation depending on their composition and abundance [75]. The lack of a significant interaction between pH and salinity in the RSM model may reflect the independent contributions of surface charge and EPS to sedimentation dynamics. Incorporating direct assessments of zeta potential and EPS in future studies would provide deeper insight into the mechanistic basis of species-specific sedimentation responses under varying environmental conditions [76, 77].

The economic analysis further underscores the scalability and practicality of the optimized sedimentation approach. While centrifugation remains one of the most efficient harvesting methods, its high energy consumption—averaging 5.8 kWh per m^3^ at a rate of $0.33/kWh—results in harvesting costs of up to $4.84 per kilogram of biomass [78–80]. In contrast, the optimized sedimentation strategy demonstrated in this study, using minimal chemical inputs yielded a cost as low as $0.71–1.01 per kilogram, depending on the species [81–84]. These findings represent an estimated 77–79% reduction in harvesting costs, a significant step toward economic feasibility for large-scale microalgal bioprocessing.

The automated cell counting method proved to be a highly efficient and reliable tool for assessing cell number, size, and morphology. This approach eliminated the need for manual counting, which is not only time-consuming but also prone to variability due to human error [19, 21]. The validation results demonstrate the high reliability of this method and a near-perfect alignment between the automated counts and manual validation. These findings underscore the method's credibility in providing accurate results with minimal human labor involvement. This is further supported by the tightly clustered data points around the regression line, underscoring the high agreement between the automated and manual methods. While the residual plot highlighted stable variability for lower cell counts, it also indicated a slight increase in variability at higher counts, suggesting minor heteroscedasticity that could result from overlapping cells or image noise at higher densities [85, 86]. Nonetheless, the normal probability plot confirmed that the residuals followed an approximately normal distribution, validating the model's robustness and predictive accuracy. Beyond its accuracy, the automated process significantly reduced the time and effort required for data analysis. Conventional manual counting methods, while effective, are labor-intensive and can introduce subjective bias. In contrast, our approach offered a streamlined, reproducible, and accessible alternative that can be readily adopted without the need for specialized machine learning models or proprietary software. This makes it a valuable addition to the toolkit of researchers working with similar cell quantification challenges.

Similarly, the non-invasive photographic method for monitoring sedimentation efficiency demonstrated its efficacy in capturing real-time dynamics without disturbing the experimental setup. By utilizing time-lapse imaging and analyzing changes in green color intensity over time, this method provided a precise and robust means to quantify sedimentation efficiency. The high correlation between photographic (SE_GCI_) and manual optical density (SE_OD_) measurements highlights the accuracy of this technique. The residual plot demonstrated consistent fluctuations, indicating homoscedasticity and the absence of systematic bias [87], while the normal probability plot showed that residuals were reasonably normally distributed, with only minor deviations at the extremes. These results validate the precision and consistency of the photographic method in capturing sedimentation efficiency over time. An added advantage of this approach lies in its non-invasive nature, which allows for continuous monitoring of sedimentation columns without physical interference. This eliminated potential disruptions to the sedimentation process and enabled accurate tracking of temporal changes. Traditional methods, such as optical density measurements, often require sample withdrawal, which can alter the experimental conditions. In contrast, the photographic approach ensured the integrity of the sedimentation process while delivering high-resolution data.

While this study demonstrates promising results, several limitations should be acknowledged. The experiments were conducted at lab scale using only two green microalgae species, which may limit generalizability across broader taxa. Additionally, while the non-invasive photographic method proved accurate and consistent under controlled indoor conditions, its application in outdoor or open pond systems may present challenges due to fluctuations in ambient lighting. Ensuring stable illumination for image-based analysis in such environments would require additional control systems or calibration strategies. Nonetheless, the method’s low cost, real-time monitoring capability, and compatibility with automated workflows make it highly promising for industrial-scale use, particularly in enclosed photobioreactors or integrated harvesting systems. Future work should assess its performance under dynamic environmental conditions and explore solutions to support deployment in less-controlled cultivation settings.

The findings of this study hold significant implications for large-scale algal bioprocessing. By establishing a robust and low-cost framework for sedimentation-based harvesting, this work advances sustainable alternatives to energy-intensive and chemically invasive methods. By minimizing energy inputs and avoiding flocculant-derived biomass contamination, the optimized sedimentation approach aligns with key priorities of the emerging bioeconomy, including low-impact production, renewable resource valorization, and circular process integration. When combined with high sedimentation efficiency and real-time, non-invasive monitoring, the approach lays a strong foundation for scalable and cost-effective downstream operations. These attributes position sedimentation not only as a viable replacement for traditional harvesting techniques but also as a strategic enabler for climate-conscious biorefineries and industrial-scale algae-based systems. Future research should explore the integration of automated sedimentation systems into continuous production pipelines and evaluate their performance under real-world conditions to bridge the gap between laboratory optimization and commercial deployment.

## Conclusions

This study advances sustainable microalgal harvesting by integrating response surface methodology (RSM) with a novel, non-invasive photographic imaging technique for sedimentation assessment. *C. sorokiniana* and *M. convolutum* demonstrated distinct growth and biochemical profiles, with BBM supporting optimal productivity for both. Sedimentation efficiency was strongly influenced by cell morphology, biochemical traits, and environmental conditions such as pH and salinity. The optimized process achieved high recovery rates (up to 96.14%) while reducing harvesting costs by up to 79% compared to centrifugation. The automated imaging methods enabled accurate, high-throughput sedimentation monitoring and validated its strong correlation with conventional techniques. These innovations offer a scalable, low-energy solution that supports bioeconomy goals and contributes to climate change mitigation by improving the economic and environmental feasibility of algae-based biofuels, bioplastics, and bioproducts.

## Data Availability

No datasets were generated or analysed during the current study.
